# Multi-variant GWAS and genomic prediction dissect the genetic architecture underlying tomato fruit weight

**DOI:** 10.1007/s00122-026-05326-2

**Published:** 2026-09-05

**Authors:** Yasin Topcu, Alper Adak, Halim Can Kayikci, Serkan Aydin, Kubra Yildiz, Alexis Ramos, Denise M. Tieman, Sofia Visa, Esther van der Knaap, Manoj Sapkota

**Affiliations:** 1 Batı Akdeniz Agricultural Research Institute, 07100 Antalya, Türkiye; 2 Institute of Plant Breeding, Genetics and Genomics, University of Georgia, Athens, GA 30602 USA; 3 Horticultural Sciences Department, University of Florida, Gainesville, FL 32611 USA; 4 Department of Mathematics and Computer Science, The College of Wooster, Wooster, OH 44691 USA; 5 Department of Horticulture, University of Georgia, Athens, GA 30602 USA; 6 Department of Horticulture, University of Kentucky, Lexington, KY 40546-0091 USA

## Abstract

**Key message:**

Tomato fruit weight is primarily controlled by stable genetic effects and domestication-/improvement-associated loci, including prominent chromosome 5 signals, and integrating SNP, INDEL, and SV diversity improves candidate-locus discovery, biological interpretation, and genomic prediction across environments.

**Abstract:**

Tomato fruit weight (fw) is a major breeding target shaped by domestication, crop improvement, and environmental variation. We investigated the genetic architecture and genomic predictability of fw in the Varitome population representing the tomato domestication continuum, including *Solanum pimpinellifolium* (SP), *S. lycopersicum* var. *cerasiforme* (SLC), and cultivated *S. lycopersicum* (SLL). Genome-wide SNPs, insertions and deletions (INDELs), and structural variants (SVs) were analyzed individually and in combination to assess their contributions to association mapping, candidate locus discovery, and genomic prediction. Population structure based on all three variant classes clearly separated the domestication groups. Genome-wide association analyses identified 15 SNP, 10 INDEL, and 10 SV loci significantly associated with fw across environments. Several associations co-localized with known fw genes, including *fw2.2/CNR*, *fw3.2/KLUH*, *fw11.3/CSR*, *lc/WUSCHEL*, and *fas/CLAVA3* supporting the biological relevance of the detected signals. Chromosome (Chr) 5 was particularly notable, with a 390-bp deletion at chr5_45,551,023 detected across all four environments and an INDEL at chr5_55,361,233 detected across three environments, suggesting stable improvement-associated candidate loci for fw variation validation. Multiple loci exhibited clear allele-frequency shifts from SP through SLC to SLL, consistent with selection during domestication and improvement, whereas others were detected almost exclusively in cultivated germplasm, suggesting more recent breeding-associated origins. Fw displayed strong genetic control, with genotype explaining more than 92% of the phenotypic variance and only minor contributions from environment and genotype-by-environment interactions. Five genomic prediction models based on SNPs, INDELs, SVs, their combinations, and genotype-by-environment interaction kernels were evaluated using four cross-validation scenarios (CV1, CV2, CV0, and CV00). Prediction accuracy was influenced more strongly by validation scenario and prediction algorithm choice than by marker class. Partial Least Squares (PLS) and Bayesian Genomic Linear Regression (BGLR) consistently outperformed Random Forest (RF) and Deep Learning (DL) models, particularly under scenarios involving untested genotypes and environments. INDEL-based models frequently achieved the highest accuracies under the most stringent prediction scenarios, although differences among marker classes were generally modest. Integrating multiple classes of genomic variation improved biological interpretation, enabled the identification of domestication- and improvement-associated loci, and provided accurate prediction across environments and genetic backgrounds. Together, these results demonstrate that tomato fw is governed primarily by stable additive genetic effects and highlight the value of multi-variant genomic approaches for both genetic dissection and genomic-assisted breeding.

**Supplementary Information:**

The online version contains supplementary material available at https://doi.org/10.1007/s00122-026-05326-2.

## Introduction

Tomato (*Solanum lycopersicum*) is one of the most important horticultural crops worldwide, with fw serving as a primary determinant of marketable yield and a central target in breeding programs (Tanksley 2004; van der Knaap et al. 2014). Over the past two decades, several key genes controlling fw have been identified and cloned in tomato, including *CELL NUMBER REGULATOR/FRUIT WEIGHT QTL 2.2* (*CNR/FW2.2*), tomato ortholog of *KLUH/CYP78A5/FRUIT WEIGHT QTL 3.2* (*SlKLUH/FW3.2)*, *CELL SIZE REGULATOR/FRUIT WEIGHT QTL 11.3* (*CSR/FW11.3*), tomato *WUSCHEL/LOCULE NUMBER* (*SlWUS*/*LC*), and tomato ortholog of *CLAVATA3/FASCIATED* (*SlCLV3/FAS*) (Frary et al. 2000; Muños et al. 2011; Chakrabarti et al. 2013; Xu et al. 2015; Mu et al. 2017). These loci contribute to fruit size variation through diverse biological mechanisms, such as cell division regulation and meristem organization. Despite the identification of several major fw genes, fw remains a complex quantitative trait influenced by numerous small-effect loci and genotype-by-environment (*G* × *E*) interactions (Ortiz et al. 2007). Accurate prediction of fw across diverse environments can significantly accelerate breeding cycles and facilitate the selection of high-yielding genotypes (Cappetta et al. 2020). The complexity of the evolution of fw cannot be fully understood without considering tomato’s domestication history. The crop was domesticated from a wild progenitor of *S. lycopersicum cerasiforme* (SLC) that evolved from *S. pimpinellifolium* (SP) (Razifard et al. 2020; Pereira et al. 2021). SLC further evolved into the fully cultivated *S. lycopersicum lycopersicum* (SLL). During this transition, allelic diversity declined substantially due to domestication bottlenecks and breeding selection (Doebley et al. 2006; Zhao et al. 2019; Razifard et al. 2020). While SP and SLC still retain rich genetic variation, modern cultivars show a narrower allelic base, particularly at loci governing fruit size and weight traits that most clearly differentiating wild from cultivated tomatoes. Notably, the domesticated SLL and semi-domesticated SLC tomatoes retain only about 22.6% and 53.8% of the standing genetic diversity of wild SP populations, respectively (Lin et al. 2014). Moreover, genomic analyses demonstrated that domestication and improvement sweeps encompassed multiple fw QTLs such as *fw2.2*, *fw3.2*, *fw11.3*, and *lcn2.1*, with severe reductions in nucleotide diversity at these loci, confirming that selection for larger fruits specifically narrowed allelic diversity in regions controlling fw (Lin et al. 2014). Because this loss of allelic diversity limits the availability of informative polymorphisms, accurate genomic prediction of fw requires models capable of capturing functional variants that remain segregating across domestication groups. Recent advances in next-generation sequencing, especially long-read (PacBio, Oxford Nanopore) and high-quality genome assembly methods have made it possible to capture genomic variation beyond SNPs, enabling accurate detection of INDELs and larger SVs (Della Coletta et al. 2021; Li et al. 2022, 2023; Zhou et al. 2022; Cui et al. 2025). INDELs can strongly influence gene function and expression when found in coding or promoter regions, respectively. SVs including large insertions, deletions, inversions, and duplications affect gene dosage, chromatin structure, as well as gene expression regulation by changing the promoter sequences (Nelson et al. 2017; Taylor et al. 2019; Alonge et al. 2020; Xu et al. 2023). High-quality genome assemblies of tomato have revealed tens of thousands of SVs distinguishing wild and cultivated genomes, underscoring their potential role in agronomic trait variation (Alonge et al. 2020). While SNPs capture abundant genome-wide variation, INDELs and SVs are more likely to directly affect gene function and regulation, potentially providing complementary information for both association mapping and genomic prediction. Despite this progress, the predictive contribution of INDELs and SVs either individually or in combination with SNPs remains largely unexplored for fw in tomato. Integrating these variant types into genomic models could improve prediction accuracy while providing deeper insights into the genetic and regulatory mechanisms underlying trait variation. Recent tomato pangenome studies have demonstrated that incorporating SVs into association and prediction models can recover missing heritability and reveal previously undetected causal loci (Alonge et al. 2020; Zhou et al. 2022; Li et al. 2023). Thus, integrative frameworks that combine SNPs, INDELs, and SVs with environmental covariates hold great potential not only to improve prediction accuracy but also to provide deeper biological insights into the genetic and regulatory mechanisms governing fw. Although fw is strongly genetically controlled, environmental variation can influence its expression and therefore affect prediction accuracy across locations and seasons. Genomic selection (GS) provides such a framework by using genome-wide molecular markers to estimate breeding values without extensive phenotyping in each generation (Meuwissen et al. 2001). Early GS approaches, such as genomic best linear unbiased prediction (GBLUP), relied on parametric models assuming linear additive effects. While effective in some contexts, these models often underperform when traits are influenced by epistasis and non-linear *G* × *E* interactions. To overcome these limitations, semi-parametric methods such as Reproducing Kernel Hilbert Space (RKHS) regression and non-parametric machine learning approaches, including Random Forests (RF) and gradient boosting, have been developed (Heslot et al. 2012; Montesinos-López et al. 2018). These methods are especially useful when modeling non-linear genotype-by-environment (*G* × *E*) interactions and epistatic effects, which are common in yield-related traits like fw. More recently, deep learning architectures have been incorporated into genomic prediction, showing promise in modeling the complex relationships among genetic variants, environments, and trait outcomes (Tong and Nikoloski 2021; Wang et al. 2023, 2025a). Neural networks particularly convolutional and fully connected models can automatically learn hierarchical features from large-scale marker data, offering an advantage for capturing epistasis and *G* × *E* interactions that are difficult to model explicitly (Ma et al. 2018; Montesinos-López et al. 2024). However, their relative performance compared with conventional statistical and machine learning models remains context-dependent, making systematic evaluation across environments essential.

Recent tomato pangenome and super-pangenome studies have demonstrated the importance of SV for capturing genomic diversity, resolving missing heritability, and improving association mapping for molecular, metabolite, flavor-related, and agronomic traits (Alonge et al. 2020; Zhou et al. 2022; Li et al. 2023; Sapkota et al. 2023). However, the relative contribution of SNPs, INDELs, and SVs to multi-environment genomic prediction of tomato fw remains poorly understood, particularly across the domestication continuum encompassing wild SP, semi-domesticated SLC, and cultivated SLL accessions. Moreover, little is known about how domestication and crop improvement have shaped the distribution, frequency, and predictive value of these different classes of genomic variation. To address these questions, we evaluated fw in a diverse tomato panel across four contrasting environments located in Türkiye, Spain, Georgia (USA), and Florida (USA). Genome-wide SNPs, INDELs, and SVs were used to develop five genomic prediction models: M1 (SNPs), M2 (INDELs), M3 (SVs), M4 (SNPs + INDELs + SVs), and M5 (M4 + genotype-by-environment interactions). Prediction performance was assessed using four complementary cross-validation schemes representing tested and untested genotypes and environments (CV1, CV2, CV0, and CV00). In addition, four prediction algorithms representing distinct analytical frameworks were compared, including BGLR, PLS, RF, and DL. This framework enabled us to evaluate the relative contributions of variant class, prediction algorithm, and genotype-by-environment interactions to fw prediction while simultaneously investigating the evolutionary trajectories of associated alleles across tomato domestication.

Specifically, our objectives were to (i) identify stable fw loci using SNPs, INDELs, and SVs across multiple environments, (ii) characterize allele-frequency shifts associated with domestication and crop improvement across SP, SLC, and SLL, (iii) compare the predictive value of different classes of genomic variation, and (iv) evaluate the performance of alternative genomic prediction algorithms under breeding-relevant scenarios involving tested and untested genotypes and environments. By integrating GWAS, evolutionary analyses, and multi-variant genomic prediction, this study provides a comprehensive framework for understanding the genetic architecture of fw and accelerating genomic-assisted breeding in tomato.

## Material and methods

### Population structure, growth regions, and statistical design

A total of 166 tomato accessions from the Varitome collection were selected for this study, representing a broad spectrum of genetic diversity within the *Solanum lycopersicum* species complex. This panel included 28 accessions of wild *S. pimpinellifolium* (SP), 117 accessions of the semi-domesticated *S. lycopersicum* var. *cerasiforme* (SLC), and 21 accessions of the fully domesticated and ancestral *S. lycopersicum* var. *lycopersicum* (SLL), as described by Mata-Nicolás et al. (2020) and Razifard et al. (2020). We acknowledge that the Varitome panel was not equally balanced across domestication groups, with SLC accessions more highly represented than SP or SLL. This imbalance may influence species-level allele-frequency comparisons and the power to detect within-group allelic effects, particularly in the smaller SP and SLL groups. Therefore, allele-frequency trajectories across SP, SLC, and SLL were interpreted as descriptive patterns consistent with domestication history, rather than as formal tests of selection. The population was evaluated across multiple environments using a randomized complete block design (RCBD). Trials were conducted in Florida and Georgia (USA), where up to three plants of the same accession were planted in two replicates, and in Antalya (Türkiye) and Valencia (Spain), where only one replicate with up to eight plants per accession was grown (Mata-Nicolás et al. 2020; Pereira et al. 2021; Kayikci et al. 2025). This multi-site, RCBD-based design provided balanced replication across contrasting agroecological regions, enabling the assessment of both main genetic effects and genotype-by-environment interactions for fw. To characterize the genetic diversity and population structure of the panel, principal component analysis (PCA) was conducted using genome-wide SNP markers. SNPs were first subjected to linkage disequilibrium (LD) pruning using an LD threshold of 0.2, a minor allele frequency threshold of 0.01, and a maximum missing data rate of 10%. Following filtering and LD pruning, 3,420 SNPs were retained from the original 1,441,362 markers and used for PCA. The analysis was performed using the SNPRelate package in R (Zheng et al. 2012), and the first two principal components were used to visualize genetic relationships among accessions and assess clustering patterns among SP, SLC, and SLL groups.

### Statistical models for phenotypic analysis

Prior to analysis, fw was normalized by applying a base-10 logarithmic transformation of the form $\log_{10} \left( {{\mathrm{FW}} + 1} \right)$ to stabilize variance and improve normality. Phenotypic data were then analyzed using both single-environment and multi-environment models. For each environment, we fitted a linear model of the form

$$y_{ijk} = {\upmu } + P_{i} + R_{j} + {\upvarepsilon}_{ijk}$$

where $y_{ijk}$ is the observed fw of the $i - {\mathrm{th}}$ pedigree in the $j - {\mathrm{th}}$ replication, μ is the overall mean, $P_{i}$ is the fixed effect of pedigree, $R_{j}$ is the random effect of replication, and $\varepsilon_{ijk}$ is the residual error. From this model, best linear unbiased predictions (BLUPs) were obtained for each pedigree in each environment and subsequently used as inputs for genomic prediction.

To jointly evaluate the entire dataset, a multi-environment linear mixed model was implemented as

$$y_{ijkl} = {\upmu } + G_{i} + E_{j} + \left( {G \times E} \right)_{ij} + R\left( E \right)_{jk} + {\upvarepsilon}_{ijkl}$$

where $y_{ijkl}$ is the observed trait value of the $i - {\mathrm{th}}$ genotype in the $j - {\mathrm{th}}$ environment and $k - {\mathrm{th}}$ replication, $G_{i} \sim N\left( {0,\sigma_{{\mathrm{G}}}^{2} } \right)$ is the fixed genetic effect, $E_{j} \sim N\left( {0,\sigma_{{\mathrm{E}}}^{2} } \right)$ is the random environmental effect, $(G \times E)_{ij} \sim N\left( {0,\sigma_{{{\mathrm{GE}}}}^{2} } \right)$ is the random genotype-by-environment interaction, $R_{k\left( j \right)}$ is the random replication nested within environment, and $\varepsilon_{ijkl} \sim N\left( {0,\sigma_{\varepsilon }^{2} } \right)$ is the residual error. The model was fitted using restricted maximum likelihood (REML) in the lmer() function of the lme4 package (Bates et al. 2015). The final model converged successfully (REML criterion at convergence = 290.5), and no convergence or singularity warnings were detected, indicating stable parameter estimation. The same mixed model was also fitted by treating genotype as a random effect to estimate the genetic variance component. This formulation allowed partitioning of the phenotypic variance into genetic ($\sigma_{{\mathrm{G}}}^{2}$), environmental ($\sigma_{{\mathrm{E}}}^{2}$), and interaction ($\sigma_{{{\mathrm{GE}}}}^{2}$) components. Based on these estimates, *H*^2^ across environments was calculated as

$$H^{2} = \frac{{{\upsigma}_{{\mathrm{G}}}^{2} }}{{{\upsigma}_{{\mathrm{G}}}^{2} + \frac{{{\upsigma}_{{{\mathrm{GE}}}}^{2} }}{{n_{{\mathrm{E}}} }} + \frac{{{\upsigma}_{{\upvarepsilon }}^{2} }}{{n_{{\mathrm{E}}} \cdot n_{{\mathrm{R}}} }}}}$$

where $n_{{\mathrm{E}}}$ is the number of environments and $n_{{\mathrm{R}}}$ is the number of replications per environment. This parameter reflects the proportion of total phenotypic variance attributable to genetic effects, adjusted for environmental and interaction variances.

### Variant discovery and genome-wide association mapping

Previously generated Illumina NextSeq 500 paired-end sequencing data (150 bp, 300-cycle chemistry) were retrieved from the NCBI Sequence Read Archive (SRP150040; BioProject PRJNA454805) and processed following the pipeline described by Kayikci et al. (2025). The datasets are publicly accessible through the NCBI Sequence Read Archive (SRA accession SRP150040; BioProject PRJNA454805) and have been reported in earlier investigations (Razifard et al. 2020; Sapkota et al. 2023; Topcu et al. 2025). Briefly, read quality assessment and filtering were performed using FastQC (v0.15.3) and Trimmomatic (Bolger et al. 2014), after which high-quality reads were aligned to the *Solanum lycopersicum* Heinz 1706 reference genome (SL4.0) using SpeedSeq (v0.1.2). In this study, INDELs and SVs were treated as operationally distinct marker classes based primarily on their variant-calling approach. INDELs were identified using the GATK (v3.8–1) small-variant calling pipeline, whereas SVs were identified independently using LUMPY (v0.2.13) and merged using SURVIVOR. This distinction follows the previously published Varitome variant-calling workflow (Pereira et al. 2021). Briefly, SNPs and small INDELs were extracted using GATK SelectVariants after joint genotyping. Raw SNPs were retained using MQ > 40, QD > 2, FS < 60, MQRankSum > − 12.5, and ReadPosRankSum > − 8. Raw INDELs were retained after excluding variants that failed any of the following hard-filtered thresholds: QD < 2, FS > 200, and ReadPosRankSum < − 20, and variants with missing data in more than 10% of accessions were removed. For GWAS, variants were further filtered using MAF ≥ 0.05. Although short-read resequencing can introduce uncertainty in small-INDEL detection, particularly in repetitive or low-complexity regions, these filtering steps were applied to reduce low-confidence variant calls before association and prediction analyses. Kinship estimation and association testing were performed using TASSEL v5.2.44 (Bradbury et al. 2007) and the BLINK model implemented in GAPIT v3 (Wang and Zhang 2021), applying a minor allele frequency threshold of 0.05 and false discovery rate (FDR) correction. Manhattan and QQ plots were generated in R using ggplot2 (Wickham et al. 2016).

To clarify the INDEL size range, we calculated the length of each retained GATK-derived INDEL allele as the absolute difference between the reference and alternate allele lengths. The final filtered GATK-INDEL set ranged from 1 to 358 bp, with 99.61% of INDEL alleles being ≤ 100 bp. Thus, the INDEL marker set was overwhelmingly composed of short insertion/deletion polymorphisms, although a small number of intermediate-size alleles were also retained by the GATK small-variant pipeline. Structural variants were identified independently using LUMPY and merged with SURVIVOR. Unlike GATK-INDELs, which are detected through local haplotype-based small-variant calling, LUMPY-derived SVs are supported primarily by discordant read-pair and split-read evidence. To evaluate possible redundancy between marker classes, we compared GATK-INDEL alleles > 100 bp with the SV dataset. Only a small number (204) of overlapping variants were detected, and after final HapMap/GWAS marker filtering, none of the overlapping variants corresponded to significant SV associations. Similarly, none of the > 100 bp GATK-INDEL alleles were among the significant INDEL associations identified in the GWAS. Therefore, overlap between intermediate-size GATK-INDELs and LUMPY/SURVIVOR SVs was minimal and did not influence the reported significant GWAS loci. Because short-read variant callers can differ in their classification of intermediate-size insertion/deletion events, particularly in repetitive or low-complexity regions, we interpret GATK-INDELs and LUMPY/SURVIVOR SVs as complementary, caller-defined marker classes rather than strictly size-separated categories. This operational distinction allows the two variant classes to capture different signatures of genomic variation while acknowledging the limitations of short-read-based variant discovery.

### Linkage disequilibrium analysis

Significant SNP, INDEL, and SV loci identified in the association analyses were used as reference loci for local LD characterization. LD was evaluated using a HapMap dataset comprising approximately 3.8 million genome-wide SNP markers. Based on our previous genome-wide LD decay analysis of the Varitome tomato collection, pairwise LD (*r*^2^) declined to 0.10 at approximately 200 kb, indicating an average genome-wide LD decay distance of ~ 200 kb (Kayikci et al. 2025). Therefore, a 200-kb upstream and 200-kb downstream interval (400-kb total window) was selected around each significant association signal for local LD analysis. For two INDEL loci, chr4_13,734,554 and chr11_31,865,292, visual inspection of the LD heatmaps indicated that LD extended beyond the original 400-kb window. Therefore, these two regions were reanalyzed using an expanded 1-Mb interval (± 500 kb) to better capture the local LD structure. Genotype data were converted to numerical format using GAPIT, and monomorphic markers were removed prior to analysis. Pairwise LD (*r*^2^) among all polymorphic markers within each interval was estimated using the LDheatmap package in *R* (Shin et al. 2006). When the exact genomic position of a significant variant was not represented in the HapMap dataset, the nearest available HapMap marker was used as the reference marker for LD estimation. The resulting LD patterns were used to characterize the extent of local LD surrounding each significant association signal. For each locus, the upstream and downstream boundaries of the LD interval were defined as the genomic positions at which LD with the reference marker decayed below an *r*^2^ threshold of 0.20 (Delourme et al. 2013; Li et al. 2014; Vos et al. 2017). If LD did not decline below *r*^2^ = 0.20 within the analyzed interval, the boundary was assigned to the outermost polymorphic marker available in that direction. These LD intervals were used to provide local genomic context for significant association signals rather than to define fine-mapped causal intervals.

### Genomic heritability estimation by marker type

To address the contribution of each marker class to the genetic variance captured by the prediction models, genomic heritability was estimated separately for SNP, INDEL, and SV marker datasets using Bayesian genomic models implemented in the BGLR package. Marker-specific genomic relationship matrices were constructed independently and matched with log-transformed fw BLUP values by pedigree name. For each marker class, an RKHS model was fitted with environment included as a fixed effect and the marker-specific genomic relationship matrix (GRM) used to model the genomic effect. Genomic heritability was calculated as $h_{g}^{2} = \frac{{\sigma_{g}^{2} }}{{\sigma_{g}^{2} + \sigma_{e}^{2} }}$ where *σ*^2^g and *σ*^2^e are the genomic and residual variance components, respectively. Models were run for 6,000 MCMC iterations with the first 1,000 iterations discarded as burn-in.

### Genomic prediction models

In this study, five prediction models (M1 to M5) were developed to evaluate the contribution of different types of genomic data and their interaction with the environment using kernel-based methods within a Bayesian framework.

#### *Model 1 (M1): SNP-based genomic prediction*

This baseline model includes fixed effects for the environment and a single random effect based on SNP markers:

$$y = X_{{{\mathrm{ENV}}}} \beta_{{{\mathrm{ENV}}}} + u_{{{\mathrm{SNP}}}} + \varepsilon$$

where $X_{ENV}$ represents the environmental covariates, and $\beta_{ENV}$ are their associated effects modeled using Bayesian ridge regression (BRR). The term $u_{{{\mathrm{SNP}}}} \sim N\left( {0,K_{{{\mathrm{SNP}}}} \sigma_{u}^{2} } \right)$ captures the random effect based on the SNP-derived relationship matrix $K_{{{\mathrm{SNP}}}}$, and $\varepsilon \sim {\mathrm{N}}\left( {0,{\upsigma}_{\varepsilon }^{2} {\mathrm{I}}} \right)$.

#### *Model 2 (M2): INDEL-based genomic prediction*

This model replaces the SNP kernel with a kernel derived from INDEL markers to evaluate the predictive power of INDEL variation:

$$y = X_{{{\mathrm{ENV}}}} \beta_{{{\mathrm{ENV}}}} + u_{{{\mathrm{INDEL}}}} + \varepsilon$$

with $$u_{{{\mathrm{INDEL}}}} \sim N\left( {0,K_{{{\mathrm{INDEL}}}} \sigma_{u}^{2} } \right)$$.

#### *Model 3 (M3): SV-based genomic prediction*

Similar to M2, this model incorporates only the SV-based kernel:

$$y = X_{{{\mathrm{ENV}}}} \beta_{{{\mathrm{ENV}}}} + u_{{{\mathrm{SV}}}} + \varepsilon$$

where $$u_{{{\mathrm{SV}}}} \sim N\left( {0,K_{{{\mathrm{SV}}}} \sigma_{u}^{2} } \right)$$.

#### *Model 4 (M4): combined additive genomic kernels (SNP* + *INDEL* + *SV)*

This model integrates all three genomic sources (SNP, INDEL, and SV) simultaneously to model their additive contributions:

$$y = X_{{{\mathrm{ENV}}}} \beta_{{{\mathrm{ENV}}}} + u_{{{\mathrm{SNP}}}} + u_{{{\mathrm{INDEL}}}} + u_{{{\mathrm{SV}}}} + \varepsilon$$

Each random effect follows its own kernel-based distribution,

#### *Model 5 (M5): full genomic* × *environment interaction model*

This comprehensive model captures both additive and interaction effects for all three genomic marker types:

$$y = X_{{{\mathrm{ENV}}}} \beta_{{{\mathrm{ENV}}}} + u_{{{\mathrm{SNP}}}} + u_{{{\mathrm{SNP}} \times {\mathrm{ENV}}}} + u_{{{\mathrm{INDEL}}}} + u_{{{\mathrm{INDEL}} \times {\mathrm{ENV}}}} + u_{{{\mathrm{SV}}}} + u_{{{\mathrm{SV}} \times {\mathrm{ENV}}}} + \varepsilon$$

Each interaction term is modeled with its corresponding interaction kernel, such as $u_{{{\mathrm{SNP}} \times {\mathrm{ENV}}}} \sim N\left( {0,K_{{{\mathrm{SNP}} \times {\mathrm{ENV}}}} \sigma_{u}^{2} } \right)$, allowing for a detailed representation of both additive and *G* × *E* components. This suite of models provides a flexible and layered framework to evaluate the predictive value of different genomic data sources and their interactions with the environment in complex trait prediction.

### Cross-validation scenarios for genomic prediction

To evaluate the performance of the five genomic prediction models based on SNPs, INDELs, and SVs, we employed four cross-validation (CV) scenarios that reflect different breeding and evaluation contexts. CV1 corresponds to predicting *tested genotypes in tested environments*, essentially benchmarking prediction accuracy within familiar trial conditions. CV2 reflects prediction of *untested genotypes in tested environments*, simulating the common breeding task of evaluating new materials under environments already represented in the training set. CV0 evaluates *tested genotypes in untested environments*, capturing the capacity of models to generalize across environments for known genetic materials. Finally, CV00 represents the most stringent case, where *untested genotypes are predicted in untested environments*, combining both genetic and environmental novelty.

For each scenario, the dataset was randomly partitioned into 70% training and 30% testing sets, and this procedure was repeated 100 times to reduce sampling bias and ensure robust estimates. Predictive ability was quantified as the Pearson correlation coefficient,

$$\rho = {\mathrm{cor}}\left( {y_{{{\mathrm{observed}}}} ,y_{{{\mathrm{predicted}}}} } \right),$$

where $y_{{{\mathrm{observed}}}}$ are the adjusted phenotypes (BLUPs from single-environment models or values from the multi-environment model) and $y_{{{\mathrm{predicted}}}}$ are the genomic predictions. Prediction accuracies were summarized across iterations as mean correlation values with their associated standard deviations. These CV frameworks are critical because they span the spectrum from evaluating model fit in familiar conditions (CV1) to assessing prediction in the most challenging case of simultaneous genetic and environmental novelty (CV00). Particular emphasis was placed on SLC and SLL, as these groups represent the semi-domesticated and cultivated stages of the tomato domestication continuum. By examining predictive ability within these groups across increasingly stringent CV scenarios, we aimed to assess how well genomic prediction models capture the transition from intermediate forms to modern cultivated tomato.

### Machine learning and Bayesian prediction algorithms

To evaluate the robustness of genomic prediction across different statistical learning frameworks, five prediction algorithms were applied to each genomic model (M1–M5): Bayesian Genomic Linear Regression (BGLR), Partial Least Squares Regression (PLS), Random Forest (RF), and Deep Learning (DL). All algorithms were trained using identical genomic inputs and cross-validation scenarios, allowing direct comparison of their predictive performance. Bayesian genomic prediction was implemented using the BGLR package in *R* (Pérez and de los Campos 2014). Environmental effects were fitted using Bayesian Ridge Regression (BRR), whereas genomic effects were modeled using Reproducing Kernel Hilbert Space (RKHS) regression. Genomic relationship matrices derived from SNPs, INDELs, and SVs were decomposed through eigenvalue decomposition, and the resulting eigenvectors and eigenvalues were incorporated as RKHS kernels. Models were fitted using 5,000 Markov Chain Monte Carlo (MCMC) iterations with a burn-in period of 1000 iterations, and posterior means were used as genomic predictions. Partial Least Squares regression was implemented using the SKM package in *R* (Boulesteix and Strimmer 2007; Montesinos López et al. 2023). Genomic relationship matrices were transformed into predictor variables through eigenvalue decomposition, and the resulting principal component scores were used as genomic features. PLS models were fitted using the kernel-based implementation available in SKM with predictor scaling disabled (scale = FALSE). This approach reduces dimensionality by constructing latent variables that maximize covariance between genomic predictors and fw while minimizing multicollinearity among predictors. Random Forest regression was implemented using the SKM package. Genomic predictors consisted of principal component features extracted from genomic relationship matrices together with environmental covariates. Each model was fitted using 500 regression trees with a minimum terminal node size of five observations. Predictions were obtained by averaging outputs across all trees. This ensemble-learning strategy improves predictive stability and enables the modeling of nonlinear marker effects and higher-order interactions without requiring explicit specification of interaction terms. DL models were implemented using TensorFlow and Keras3 through the *R* interface. To reduce dimensionality and computational complexity, the first 30 principal components derived from each GRM were retained as predictors. Predictor variables were standardized using training-set statistics and response values were scaled prior to model fitting. The neural network architecture consisted of an input layer followed by a fully connected hidden layer containing 10 neurons with ReLU activation, L2 regularization (*λ* = 0.01), a dropout layer with a dropout rate of 0.30, and a single-neuron output layer for regression. Models were optimized using the Adam optimizer with a learning rate of 0.001 and mean squared error loss. Training was performed for up to 100 epochs with a batch size of 16, and early stopping with a patience parameter of 20 epochs was used to reduce overfitting and restore the best-performing model weights.

### LD pruning based genomic prediction

To evaluate the effect of marker density on genomic prediction, SNP markers were filtered using pairwise LD thresholds implemented with the snpgdsLDpruning function in the SNPRelate *R* package (Zheng et al. 2012). Pairwise LD thresholds ranging from *r*^2^ = 0.1 to 0.9 (in increments of 0.1) were used to generate nine marker datasets with progressively increasing marker densities. For each marker dataset, a GRM was constructed using the same procedure described for the primary genomic prediction analyses. Each GRM was subsequently decomposed into genomic features through eigenvalue decomposition and combined with environmental fixed effects as predictors in a PLS regression model implemented in the SKM R package (Montesinos López et al. 2022, 2023). To enable a direct comparison among marker densities, the identical 100 random train–test partitions and the same four cross-validation scenarios (CV1, CV2, CV0, and CV00) used in the primary genomic prediction analyses were applied to all marker datasets. Prediction performance was evaluated using Pearson’s correlation coefficient (prediction ability), root mean square error (RMSE), mean absolute error (MAE), and prediction bias. Pearson’s correlation quantified the agreement between observed and predicted fw BLUPs, RMSE measured the average magnitude of prediction errors while giving greater weight to larger deviations, MAE represented the average absolute prediction error, and prediction bias was calculated as the mean difference between predicted and observed fw BLUPs. Bias values close to zero indicated unbiased predictions, whereas positive and negative values indicated systematic overestimation and underestimation, respectively. For each cross-validation scenario, prediction model, and algorithm, all metrics were calculated for every iteration and summarized as the mean ± standard deviation across 100 iterations.

$$r = \left( {y,{ }\hat{y}} \right)$$

$${\mathrm{RMSE}} = \sqrt {\frac{1}{n}\mathop \sum \limits_{i = 1}^{n} (y_{i} - \hat{y}_{i} )^{2} }$$

$${\mathrm{MAE}} = \frac{1}{n}\mathop \sum \limits_{i = 1}^{n} \left| {y_{i} - \hat{y}_{i} } \right|$$

$${\mathrm{Bias}} = \frac{1}{n}\mathop \sum \limits_{i = 1}^{n} (\hat{y}_{i} - y_{i} )$$

where: $y_{i}$ is the observed phenotype, $\hat{y}_{i}$ is the predicted phenotype, n is the number of observations in the testing set. Prediction performance of four algorithms (BGLR, PLS, RF, and DL) was compared across five prediction models (M1, M2, M3, M4 and M5) within each cross-validation scenario (CV1, CV2, CV0, and CV00). For each cross-validation scenario (CV1, CV2, CV0, and CV00), a linear mixed-effects model was fitted with prediction accuracy as the response variable, prediction algorithm, model, and their interaction as fixed effects, and iteration included as a random effect to account for repeated use of the same train–test partitions:

$${\mathrm{cor}} \sim {\mathrm{Algorithm}} \times {\mathrm{Model}} + \left( {1{\mathrm{Iteration}}} \right)$$

Type III analysis of variance (ANOVA) with Satterthwaite’s approximation was performed separately for each cross-validation scenario to evaluate the effects of prediction algorithm, prediction model, and their interaction. Estimated marginal means were subsequently computed, and Tukey-adjusted multiple comparisons were conducted to compare prediction algorithms within each prediction model.

## Results

### Fruit weight variation across environments

**Fig. 1:**
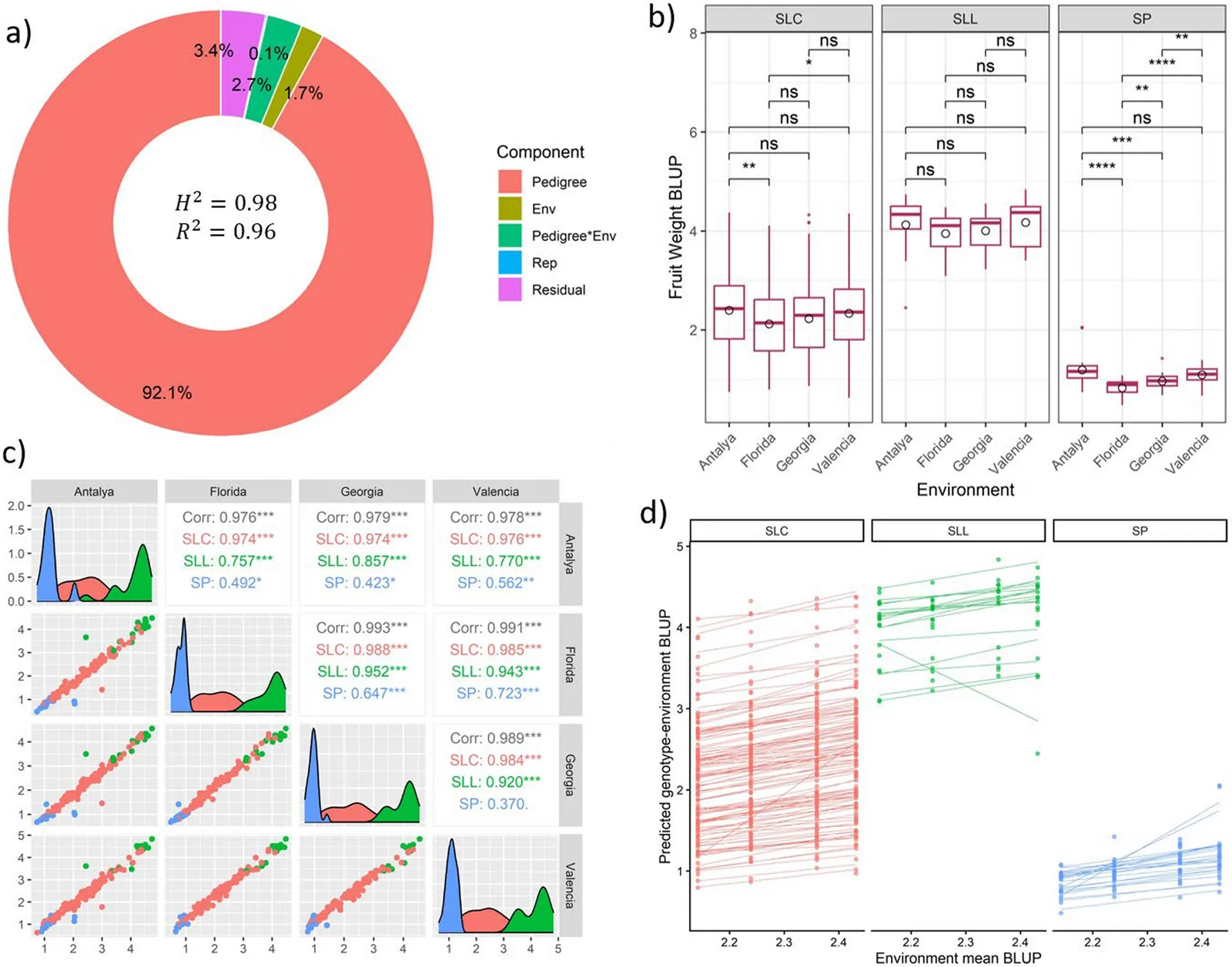
Genetic control, environmental stability, and genotype responsiveness of fw across environments. **a** Variance component analysis from a multi-environment mixed model with all effects treated as random. **b** Distribution of single-environment BLUPs for fw across four environments (Antalya, Florida, Georgia, and Valencia) within each species group. Open circles indicate species means. Pairwise comparisons among environments within species were performed using Wilcoxon rank-sum tests, with significance levels denoted as ns, *, **, ***, and ****. **c** Pearson correlations of genotype BLUPs among environments, visualized using pairwise scatterplots and density distributions. Correlation coefficients are shown for all accessions combined (Corr) and separately for SLC, SLL, and SP. SLC, SLL, and SP represent *Solanum lycopersicum* var. *cerasiforme*, *S. lycopersicum* var. *lycopersicum*, and *S. pimpinellifolium*, respectively. **d** Finlay–Wilkinson regression (reaction norm analysis) describing genotype responsiveness to environmental quality. Each line represents an individual genotype, relating its predicted genotype–environment BLUP to the environmental mean BLUP across the four environments. Steeper slopes indicate greater environmental responsiveness, whereas flatter slopes indicate greater stability across environments

Variance components analysis of multi environment model showed that genotype explained the vast majority of phenotypic variation in fw (92.1%), while environment, genotype × environment (GXE) interaction, and replication contributed only marginally. The residual accounted for 3.4% of variation. The ANOVA model fit was high (*R*^2^ = 0.96) with strong broad-sense heritability (*H*^2^ = 0.98), underscoring the strong genetic basis of the trait **(**Fig. 1a**)**. Consistent with this strong genetic control, fw distributions differed markedly among species but showed contrasting environmental responses. BLUPs revealed consistent differences among tomato species across all four environments (Antalya, Florida, Georgia, and Valencia). SLL accessions consistently exhibited the largest fw, with median BLUP values close to 4.0–4.5, whereas SLC accessions showed intermediate values (2.0–3.0) and SP accessions produced the smallest fruits (0.8–1.3). Pairwise Wilcoxon comparisons confirmed significant differences between SP and both SLL and SLC groups across most environments. Environmental effects within species were comparatively limited. Significant differences among environments were detected for SP, whereas SLL showed no significant environmental differences and SLC exhibited only minor environmental effects, suggesting greater phenotypic stability especially in SLL germplasm (Fig. 1b). Fw in SP was significantly lower in Florida compared with Antalya, Georgia, and Valencia, while additional significant differences were detected between several other environment pairs. The broader spread of pairwise significance tests in SP indicates stronger environmental effects on fw expression relative to the cultivated groups as well **(**Fig. 1b**)**. Cross-environment correlation analyses further supported these patterns. Fw showed strong overall correlations among environments, indicating stable genotype ranking. However, species-specific analyses revealed clear differences in environmental robustness. Correlation analysis demonstrated strong cross-environment stability for SLC and SLL (*r* = 0.76–0.99), while SP exhibited low or nonsignificant correlations across environments (*r* = 0.37–0.72). This indicates that SP performance was highly environment-dependent, whereas cultivated and semi-domesticated species maintained consistent relative rankings **(**Fig. 1c**)**. In the Finlay–Wilkinson regression plots, we observed distinct responses of genotypes across environmental means, reflecting varying degrees of environmental sensitivity **(**Fig. 1d**)**. SLL accessions displayed nearly parallel reaction norms with shallow slopes, highlighting strong buffering against environmental variation. SLC accessions showed moderate heterogeneity in slopes, while SP accessions exhibited steep and highly variable reaction norms, indicating strong genotype-by-environment responses. Together, these results demonstrate that fw is a strongly genetically controlled trait and that domestication has progressively reduced environmental sensitivity. Wild SP accessions retain high plasticity, whereas cultivated SLL accessions show pronounced phenotypic stability, consistent with selection for canalized fw during tomato domestication. Single-environment *H*^2^ estimates further supported the strong genetic control of fw. Because Antalya and Valencia were evaluated with one replication, environment-specific *H*^2^ could only be estimated for the replicated Florida and Georgia trials. In Florida, genotype accounted for 97.61% of the phenotypic variance, with genetic and residual variance estimates of 1.003 and 0.023, respectively, resulting in *H*^2^ = 0.989. In Georgia, genotype accounted for 95.77% of the phenotypic variance, with genetic and residual variance estimates of 0.983 and 0.042, respectively, resulting in *H*^2^ = 0.979. These results indicate that fw was highly heritable within each replicated environment, with genetic effects explaining more than 95% of the observed phenotypic variation.

### Population structure and genomic distribution of variants

**Fig. 2:**
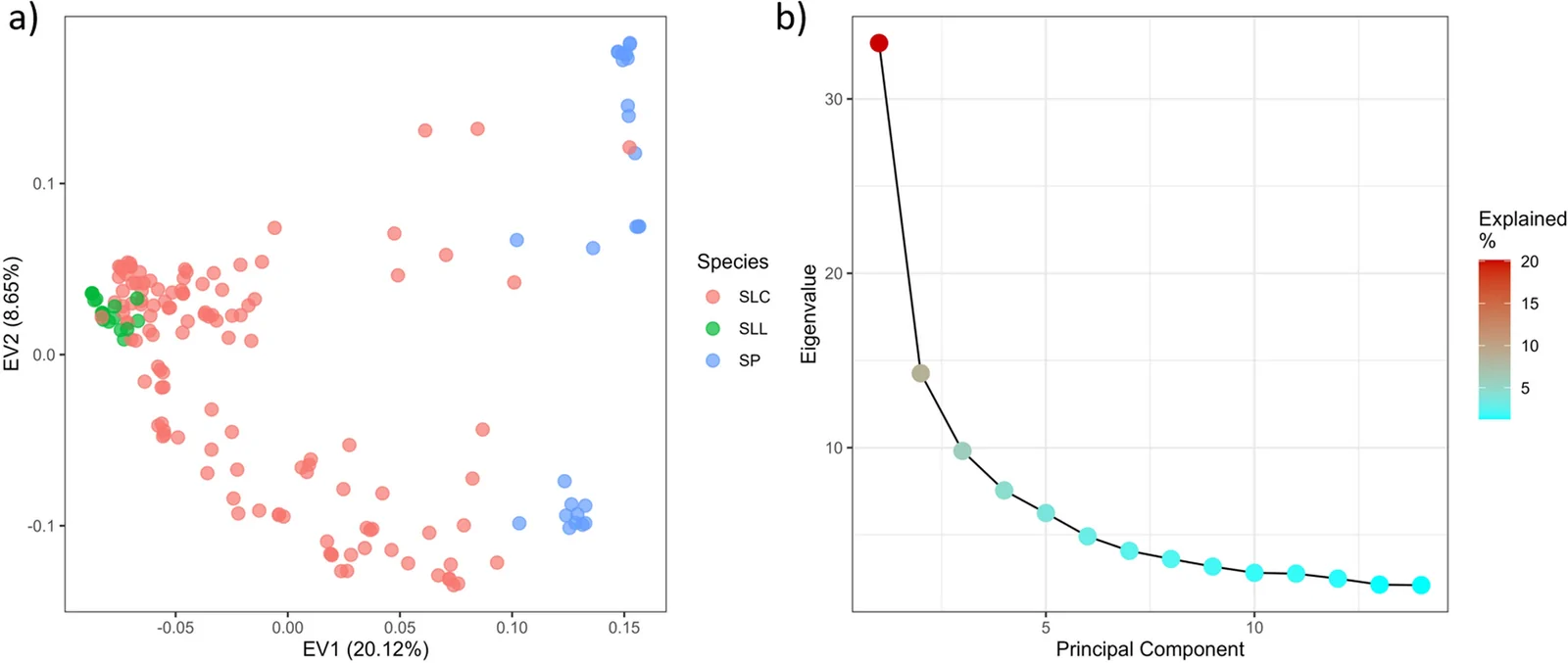
Population structure of the Varitome tomato panel based on principal component analysis (PCA) using genome-wide SNP markers. **a** Scatter plot of the first two principal components (PC1 and PC2), explaining 20.12% and 8.65% of the total genetic variation, respectively. Each point represents an accession and is colored according to species: *Solanum lycopersicum* var. *cerasiforme* (SLC, red), *S. lycopersicum* var. *lycopersicum* (SLL, green), and *S. pimpinellifolium* (SP, blue). The PCA reveals clear genetic differentiation among species, with SP accessions forming a distinct cluster from cultivated tomato groups. **b** Scree plot showing the eigenvalues of the first 14 principal components. Point colors indicate the percentage of variance explained by each component, highlighting the contribution of the leading principal components to the overall genetic variation

**Fig. 3:**
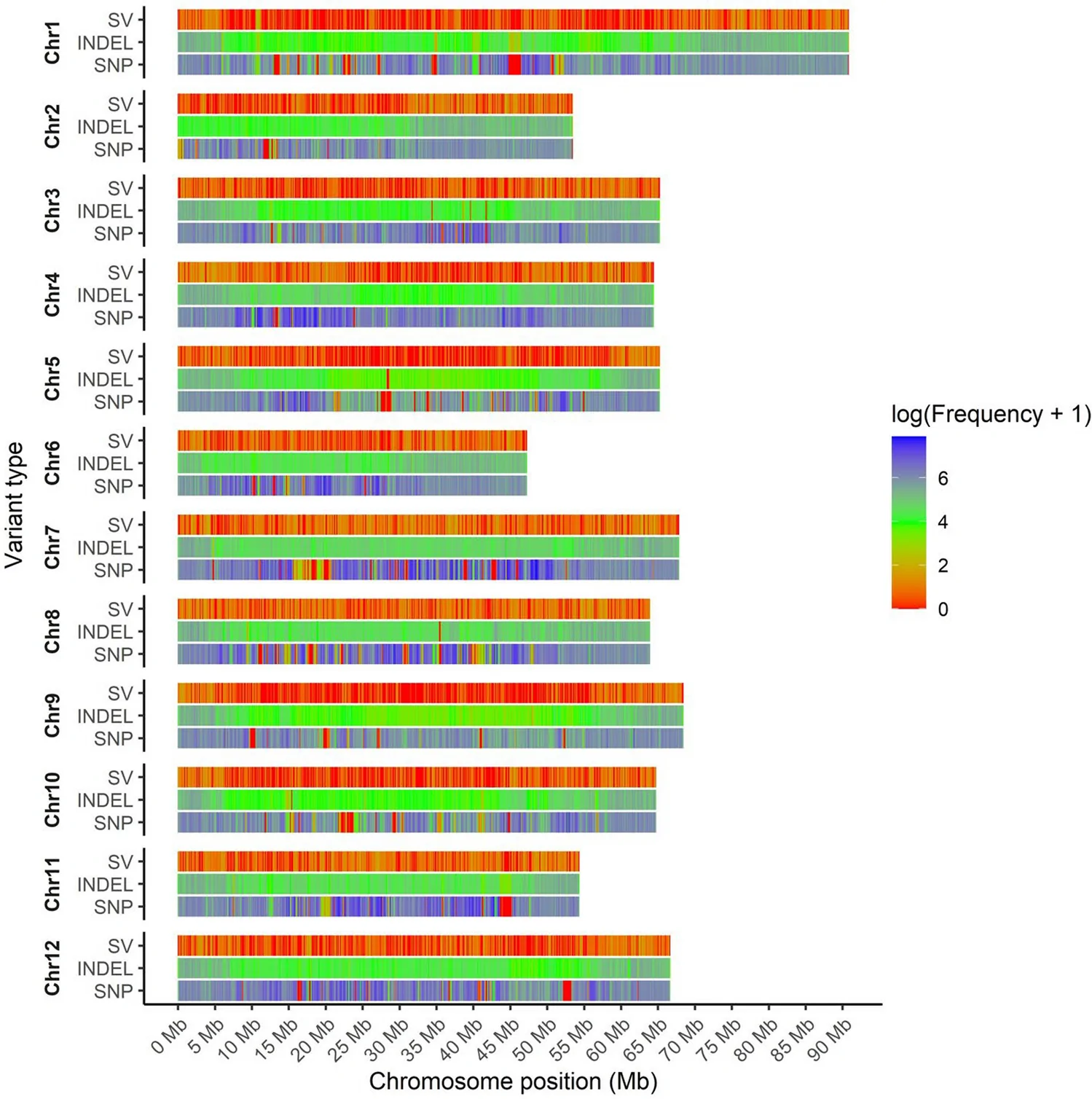
Genomic distribution of SNPs, INDELs, and SVs across the tomato genome. Heatmaps show the density of variant counts in non-overlapping 100 kb windows along Chr 1–12. Each row within a chromosome represents one variant type (SNP, INDEL, or SV), with color intensity corresponding to the log-transformed frequency of variants in that window. Warmer colors indicate regions with fewer variants, while cooler colors denote regions of higher density. This representation illustrates the uneven distribution of variants across the genome and emphasizes regions with strong accumulation or depletion of specific variant types

PCA based on SNP markers revealed clear genetic differentiation among the three tomato species groups in the Varitome panel **(**Fig. 2a**)**. The first principal component (PC1) explained 20.12% of the total genetic variation and primarily separated wild SP accessions from the cultivated (SLL) and semi-cultivated (SLC) tomato groups. The second principal component (PC2) accounted for an additional 8.65% of the variation and captured within-species diversity, particularly among SP accessions. Together, PC1 and PC2 explained 28.77% of the total genomic variation. The PCA revealed a distinct domestication gradient. SP accessions formed a clearly separated cluster characterized by broad dispersion along both principal components, indicating high levels of genetic diversity. In contrast, SLL accessions clustered tightly near the center of the plot, reflecting the reduced genetic diversity associated with domestication and modern breeding. SLC occupied an intermediate position between SP and SLL and displayed moderate dispersion, consistent with its recognized role as a transitional population between wild and cultivated tomatoes. The scree plot further demonstrated that genetic variation was concentrated in the first few principal components **(**Fig. 2b**).** PC1 and PC2 explained the majority of the variation whereas subsequent components each explained progressively smaller proportions of variation (< 6%). The rapid decline in eigenvalues after the first few components indicates that the major axes of genetic structure are largely captured by the leading principal components and are primarily driven by species-level differentiation. Overall, the PCA results demonstrate strong population structure within the Varitome panel, with clear separation of SP, SLC, and SLL accessions and a progressive reduction in genetic diversity from wild to cultivated tomato groups. The genome-wide distribution of SNPs, INDELs, and SVs revealed pronounced heterogeneity across chromosomes and among variant classes. Following filtering steps, 3,879,252 SNPs, 831,152 INDELs, and 11,447 SVs were retained. Genome-wide distributions of SNPs, INDELs, and SVs were further assessed in non-overlapping 100 kb windows along all 12 chromosomes **(**Fig. 3**)**. SNP densities showed clear chr-level differences, with mean values per 100 kb window ranging from 377 on chr 9 to 646 on Chr 7. Maximum SNP counts per window varied substantially, from 1,327 on chr 2 to 2,652 on chr 7, indicating localized regions of high polymorphism. INDEL densities followed a similar pattern but at lower overall frequencies, with chr-wide means ranging from 80 (chr 9) to 130 (chr 7) per window. Peak INDEL densities ranged from 336 on chr 5 to 428 on chr 8, suggesting chr and region-specific enrichment of small insertion–deletion events. SVs were comparatively rare and exhibited the strongest spatial heterogeneity. Mean SV frequencies per 100 kb window ranged from 0.99 on chr 9 to 1.93 on chr 7, while maximum SV counts varied from 8 on chr 5 to 25 on chr 1, consistent with the expectation that large-scale genomic rearrangements are less frequent and more spatially constrained. The total number of 100 kb windows reflected chr length, ranging from 473 bins on chr6 to 909 bins on chr1, confirming adequate genome coverage across chromosomes. Collectively, these patterns indicate that chr 7 harbors the highest overall level of genetic variation, whereas chr 9 consistently exhibits the lowest, across all three variant classes. The concordance of SNP, INDEL, and SV density patterns suggests that chr-specific differences in recombination landscape, selective constraints, and domestication-related processes have jointly shaped the current genomic architecture of variation in tomato.

### Genome-wide associations for fw across environments

**Fig. 4:**
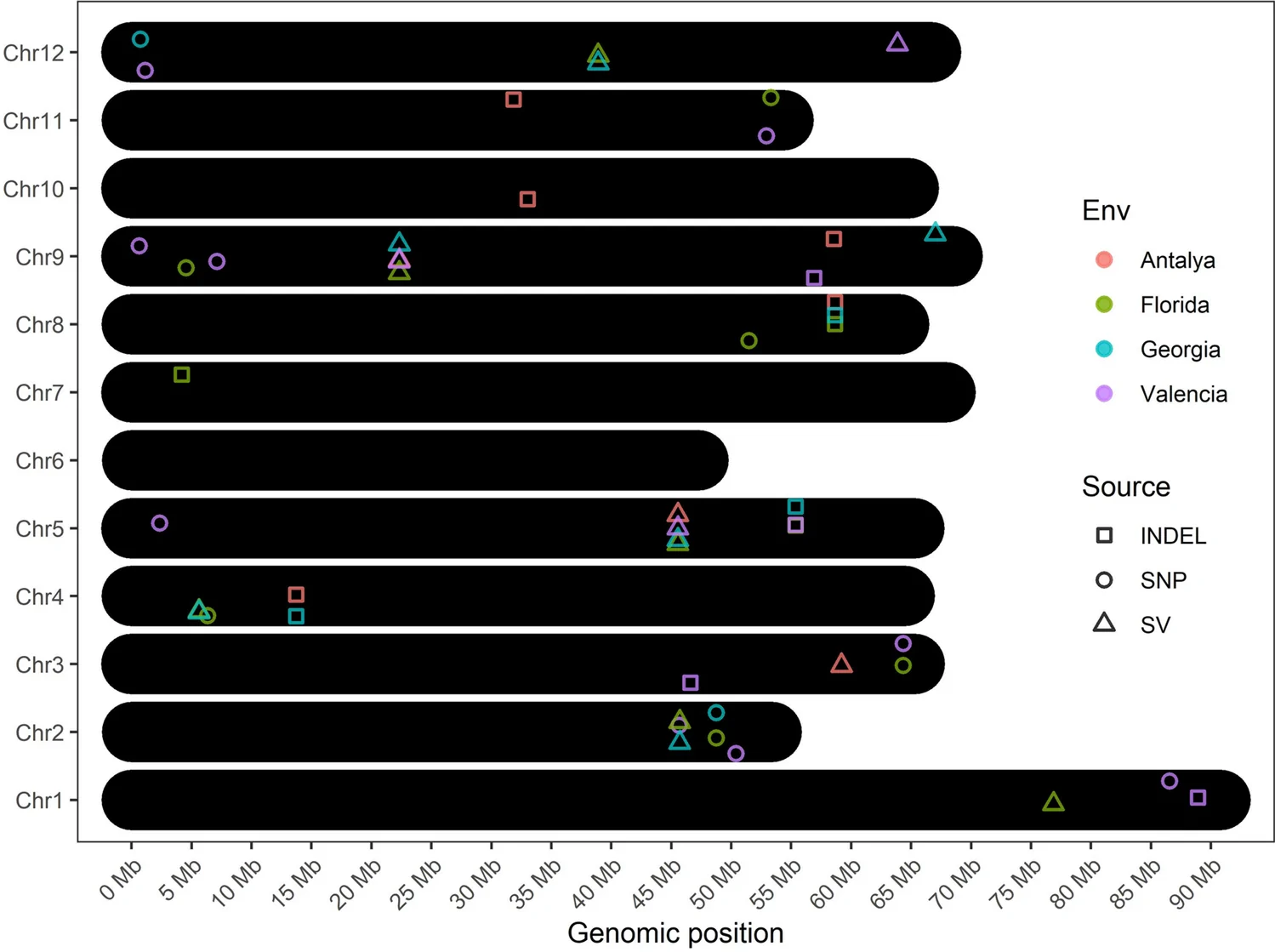
Genomic distribution of significant GWAS associations for fw across four environments using multiple variant types. Significant marker–trait associations are plotted according to their physical positions on the 12 tomato chromosomes (Chr1–Chr12). Black horizontal bars represent chromosomes scaled to their physical lengths, and the x-axis indicates genomic position (Mb). Marker shapes denote variant type (squares = INDELs, circles = SNPs, and triangles = structural variants [SVs]), while marker colors indicate the environment in which each association was detected: Antalya (red), Florida (green), Georgia (cyan), and Valencia (purple). The distribution of significant associations across chromosomes highlights both stable genomic regions detected in multiple environments and environment-specific loci contributing to fruit weight variation in tomato

GWAS analyses identified multiple loci significantly associated with fw across four independent environments (Antalya/Türkiye, Valencia/Spain, Georgia and Florida/USA), revealing both stable and environment-specific genetic signals distributed across all 12 tomato chromosomes (Fig. 4, and Supplementary Table S1). Significant associations were detected for all three variant classes SNPs, INDELs, and SVs highlighting the complex genetic architecture underlying fw. Signals were distributed across all 12 tomato chromosomes and represented by three variant classes. In total, 10 INDELs, 15 SNPs, and 10 SVs were significantly associated (Supplementary Fig. S1–S3). Genome-wide Q–Q plots for each marker class and environment are provided in Supplementary Fig. S4, allowing assessment of the distribution of observed association statistics relative to the null expectation. To further characterize these associations, local LD intervals were estimated for each significant SNP, INDEL, and SV locus using a 400-kb window centered on the significant marker. LD boundaries were defined as the positions where LD with the focal marker decayed below *r*^2^ = 0.20. Because the chr4_13,734,554 and chr11_31,865,292 INDEL loci showed extended LD across the original window, these two regions were reanalyzed using an expanded 1-Mb interval. LD boundaries were defined as the positions where LD with the focal marker decayed below *r*^2^ = 0.20, or as the outermost polymorphic marker in the analyzed interval when LD did not decay below this threshold. The resulting LD intervals, block sizes, and number of polymorphic markers within each LD block are summarized in Supplementary Table S2, and LD heatmaps for SNP, INDEL, and SV loci are shown in Supplementary Fig. S5–S7, respectively. These analyses provide local genomic context for the significant associations and support the interpretation of candidate regions surrounding stable fw loci. Several loci showed environmentally stable effects, being repeatedly detected in multiple environments. For SNP-based GWAS, a total of 17 significant marker–environment associations were detected, representing 15 unique SNP loci. Among these, two SNP loci were repeatedly detected in more than one environment, suggesting relatively stable effects on fw. The SNP at chr2_48,756,488 bp was significant in both Florida and Georgia, with –log_10_(P) values of 9.66 and 13.35, respectively. When considering broader genomic regions, a major chr 2 association spanning 45.66–50.41 Mb was detected in Florida, Georgia, and Valencia, suggesting a stable QTL across multiple environments. Similarly, the SNP at chr3_64,357,321 bp was detected in both Florida and Valencia, with –log_10_(P) values of 10.52 and 12.95, respectively. Additional recurrent signals were observed on chr 11 (52.95–53.30 Mb), where significant SNPs were detected in both Florida and Valencia. The most significant SNP association was identified at chr5_2,353,343 bp in Valencia, corresponding to an A/G polymorphism with MAF = 0.13, an estimated effect of 0.29, and a –log_10_(P) value of 18.81. Other highly significant SNP signals included chr9_7,123,848 bp in Valencia (–log_10_(P) = 17.27) and chr11_52,950,253 bp in Valencia (–log_10_(P) = 15.54). A significant SNP at chr2_50,414,767 bp (G/A; –log_10_(P) = 10.14; MAF = 0.45) was identified in the Valencia environment. This SNP is located in close proximity to the major fw gene *fw2.2/CNR*, corresponding to the *Cell Number Regulator* gene (*Solyc02g090730*), which spans chr 2_50,292,691–50,293,481 bp. The associated SNP is located approximately 121 kb downstream of *Solyc02g090730*, making *fw2.2* a plausible candidate gene underlying this association. For INDEL based GWAS, 15 significant marker–environment associations were detected, corresponding to 10 unique genomic loci. The most significant INDEL association was observed at chr9_56,914,628 bp in Valencia (–log_10_(P) = 13.17), closely followed by the environmentally stable locus at chr5_55,361,233 bp (maximum –log_10_(P) = 13.12). Three INDEL loci were repeatedly identified across multiple environments, suggesting stable effects on fw. The most consistent INDEL signal was detected at chr5_55,361,233 bp (Ref: CAGTT; Alternative: C), which was significantly associated with fw in Florida, Valencia, and Georgia, with –log_10_(P) values ranging from 8.54 to 13.12. Another stable locus was identified at chr8_58,643,238 bp (Ref: AT; Alternative: A), detected in Antalya, Florida, and Georgia, with –log_10_(P) values ranging from 9.39 to 11.25. In addition, an INDEL at chr4_13,734,554 bp (Ref: A; Alternative: AT, ATT, ATTT) was detected in Antalya and Georgia. An additional potential recurrent INDEL region was observed on chr 9, where significant signals at 56.91 Mb and 58.56 Mb were detected in Valencia and Antalya, respectively. However, because these markers showed opposite effect directions, this region may represent either a complex locus or distinct nearby environment-specific associations. The remaining five INDEL loci were environment-specific, including signals on chr 1, 3, 7, 10, and 11. For SV-based GWAS, 18 significant marker environment associations were detected, corresponding to 10 unique SV loci. Several SV loci were repeatedly identified across multiple environments, indicating stable effects on fw. The most consistent signal was a 390-bp deletion at chr5_45,551,013 bp, which was significantly associated with fw in all four environments (Antalya, Florida, Georgia, and Valencia), with –log_10_(P) values ranging from 7.31 to 18.85. A second stable SV locus, corresponding to a 1,040-bp deletion at chr9_22,330,015 bp, was detected in Antalya, Florida, and Georgia, with –log_10_(P) values ranging from 6.96 to 11.22. Additional recurrent SV loci included a 378-bp deletion at chr2_45,709,317 bp, a 9,486-bp deletion at chr4_5,630,658 bp, and a 413-bp deletion at chr12_38,911,139 bp, each detected in both Florida and Georgia. A significant SV corresponding to a 50,444-bp duplication at chr3_59,206,418 bp was identified in Antalya (–log₁₀(P) = 6.82; MAF = 0.18). This SV is located approximately 11 kb upstream of the known fw gene *fw3.2/SlKLUH* (*Solyc03g114940*; chr_03:59,217,389–59,219,730 bp), making *SlKLUH* a plausible candidate gene underlying this association. The remaining four SV loci were also environment-specific and located on chr 1, 9, and 12. Overall, the GWAS results demonstrate that fw in tomato is controlled by a combination of stable, environment-independent loci and conditional, environment-specific genetic effects. The detection of significant associations across SNPs, INDELs, and SVs underscores the importance of integrating multiple variant classes to comprehensively resolve the genetic basis of complex quantitative traits.

### INDEL, SNP and SV associated scenarios across tomato species

**Fig. 5:**
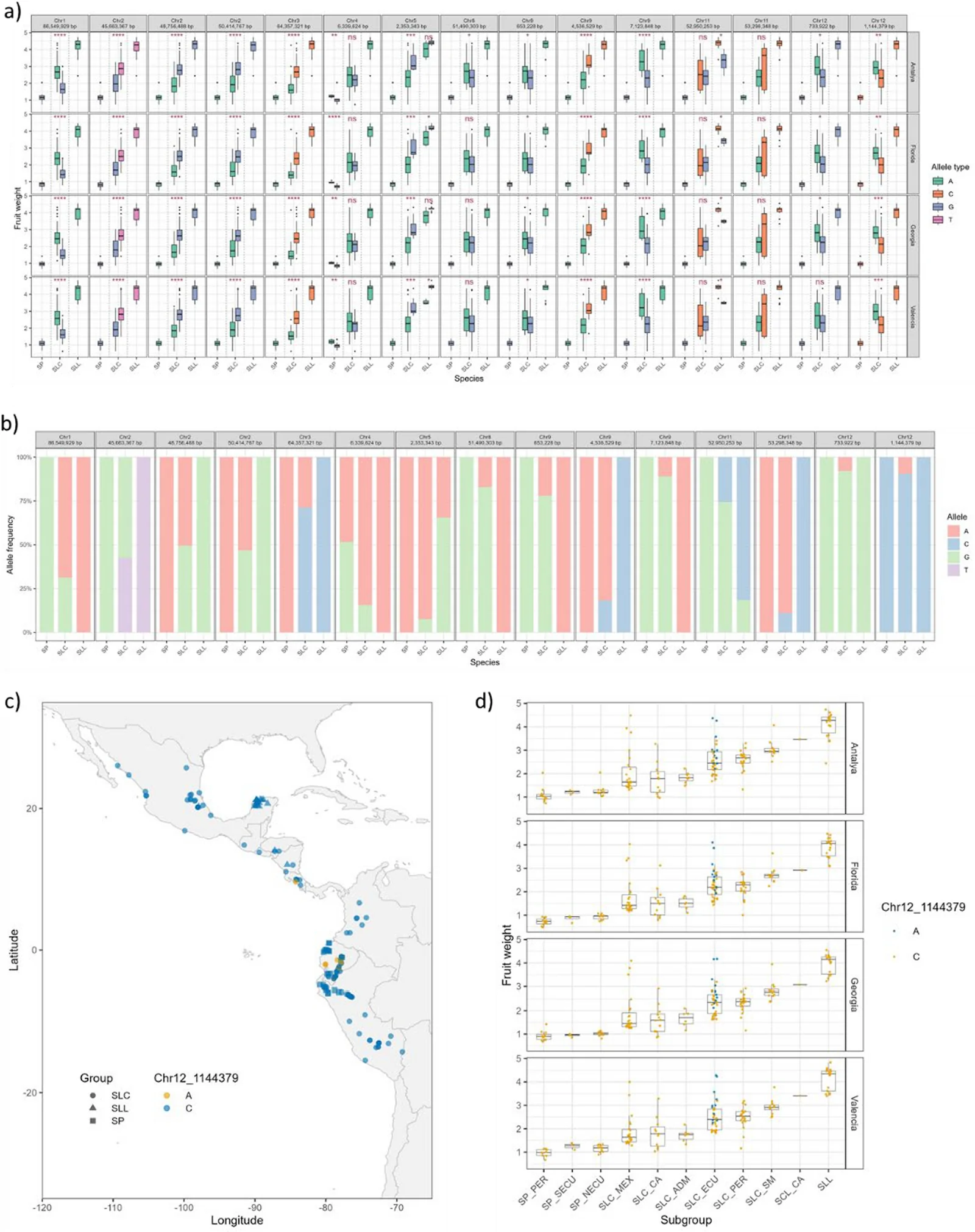
Patterns of SNP variation and their phenotypic associations across tomato species. **a** Boxplots showing the effect of GWAS-detected SNP markers on fw (BLUPs transformed) across SP, SLC, and SLL. Each panel corresponds to a significant SNP marker identified in the genome-wide association study. Different colors represent the SNP alleles. Detailed information for each SNP polymorphism, including chromosome, genomic position, allele composition, and variant annotation, is provided in Table S1. Statistical significance between allelic classes is indicated above each facet. **b** Allele frequency distribution of the same SNP markers across SP, SLC, and SLL. Different colors represent the SNP alleles. The bar chart illustrates variation in allele frequencies among tomato species and highlights allele frequency shifts associated with tomato domestication and improvement. **c** Geographic distribution of the SNP allele (Ref:C/Alternative:A) at Chr12_1,144,379 bp across the tomato germplasm collection. **d** Distribution of fw BLUPs among tomato genetic subgroups according to allelic state at Chr12_1,144,379 bp across Antalya, Florida, Georgia, and Valencia. Boxplots show subgroup-specific distributions, while points represent individual accessions

**Fig. 6:**
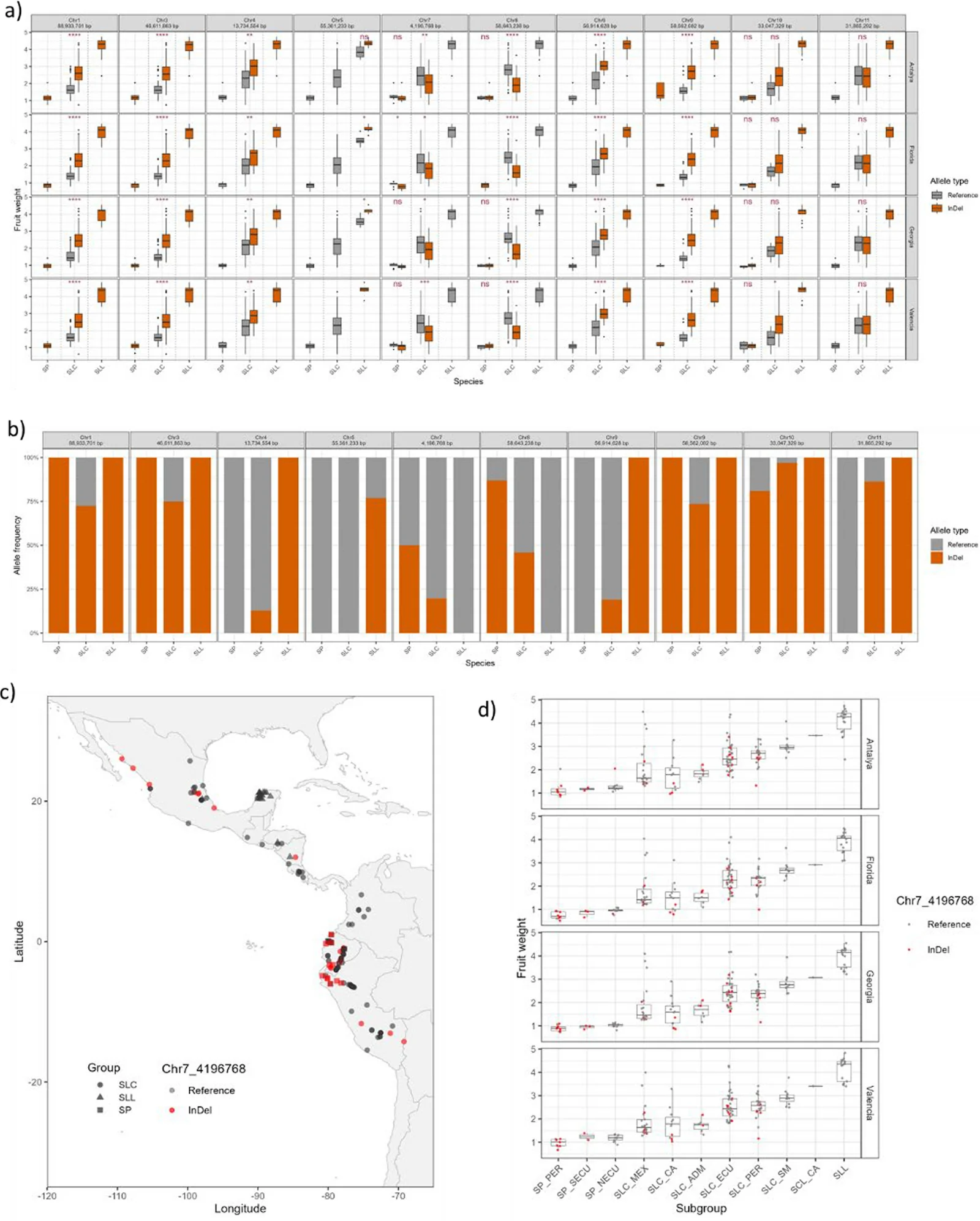
Patterns of insertion–deletion (INDEL) variation and their phenotypic associations across tomato species. **a** Boxplots showing the effect of GWAS-detected INDEL markers on fw (BLUPs transformed) across SP, SLC, and SLL). Each panel corresponds to a significant INDEL marker identified in the genome-wide association study. Different colors represent the Reference and INDEL alleles. Detailed information for each INDEL polymorphism, including chromosome, genomic position, allele composition, and variant annotation, is provided in Table S1. Statistical significance between allele classes is indicated above each facet. **b** Frequency distribution of the same INDEL markers across SP, SLC, and SLL. Different colors represent the Reference and INDEL alleles. The bar chart illustrates changes in allele frequencies among tomato species, highlighting shifts associated with tomato domestication and improvement.** c** Geographic distribution of the INDEL allele (Ref: GT, Alternative:G) at Chr7_4,196,768 bp across the tomato germplasm collection. **d** Distribution of fruit weight BLUPs among tomato genetic subgroups according to allelic state at Chr7_4,196,768 bp across Antalya, Florida, Georgia, and Valencia. Boxplots show subgroup-specific distributions, while points represent individual accessions

**Fig. 7:**
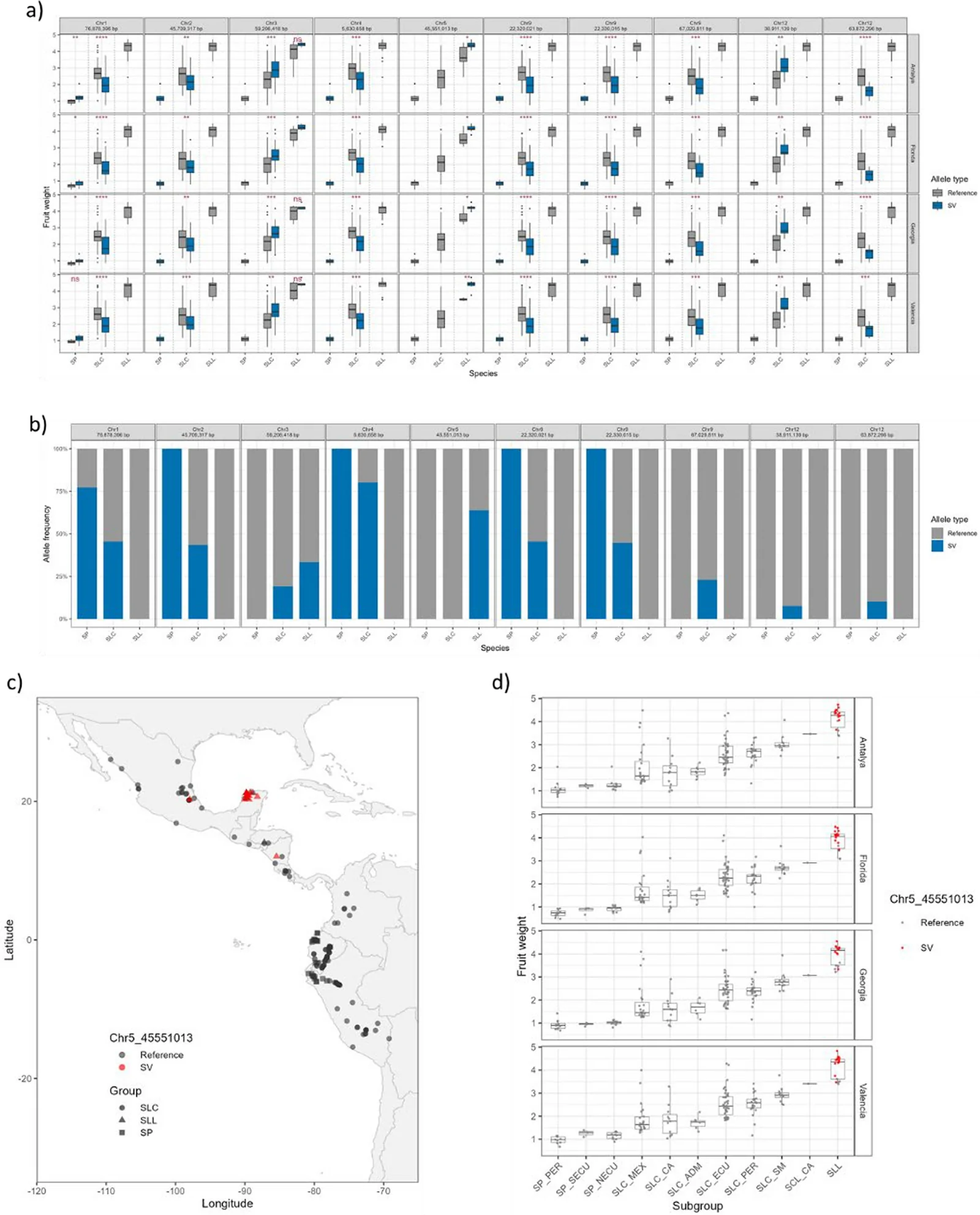
Patterns of SV and their phenotypic associations across tomato species. **a** Boxplots showing the effect of GWAS-detected SV markers on fw (BLUPs transformed) across SP, SLC, and SLL. Each panel corresponds to a significant SV marker identified in the genome-wide association study. Different colors represent the Reference and SV alleles. Detailed information for each structural variant, including chromosome, genomic position, allele composition, and variant annotation, is provided in Table S1. Statistical significance between allele classes is indicated above each facet. **b** Allele frequency distribution of the same SV markers across SP, SLC, and SLL. Different colors represent the Reference and SV alleles. The bar chart illustrates variation in SV allele frequencies among tomato species and highlights shifts associated with tomato domestication and improvement.** c** Geographic distribution of the SV allele (390 bp Deletion) at Chr5_45,551,013 bp across the tomato germplasm collection. **d** Distribution of fw BLUPs among tomato genetic subgroups according to allelic state at Chr5_45,551,013 bp across Antalya, Florida, Georgia, and Valencia. Boxplots show subgroup-specific distributions, while points represent individual accessions

Comparative analyses of SNPs, INDELs, and SVs associations revealed multiple, distinct evolutionary and phenotypic scenarios underlying fw variation across SP, SLC, and SLL (Figs. 5, 6, and 7). These patterns highlight how allelic emergence, selection, and fixation have differentially shaped fw during tomato domestication and subsequent improvement. Analysis of SNP variation revealed several allele frequency and phenotypic effect patterns **(**Fig. 5a and b**).** Across multiple loci (chr1_86,549,929 bp, chr2_45,663,367 bp, chr2_48,756,488 bp, chr2_50,414,767 bp, chr3_64,357,321 bp, chr8_51,490,303 bp, chr9_653,228 bp, chr9_4,536,529, chr9_7,123,848 and chr11_52,950,253 bp), alleles associated with increased fw were absent in SP, emerged in SLC, and subsequently became fixed in cultivated SLL, consistent with stepwise selection during domestication and improvement. Particularly noteworthy was a cluster of significant SNPs spanning 45.66–50.41 Mb on chr 2. All three loci displayed nearly identical domestication patterns, with favorable alleles absent in SP and fixed in SLL, suggesting that this region represents a major target of selection. The SNP at chr2_50,414,767 bp lies approximately 121 kb downstream of *fw2.2/Cell Number Regulator* (*Solyc02g090730*), further supporting the biological relevance of this region for fw variation. While most fw-associated loci showed progressive enhancement of favorable alleles from SP to SLC and SLL, the chr 12 loci displayed a contrasting evolutionary pattern. At chr12_733,922 bp and chr12_1,144,379 bp, the favorable alleles were absent in SP, increased in frequency within SLC accessions only collected in Ecuador, but were subsequently lost or nearly eliminated in SLL **(**Fig. 5c and d**)**. This pattern suggests that these alleles may have contributed to fw variation during early domestication but were not retained during subsequent crop improvement. The disappearance of these alleles from early cultivated germplasm indicates that selection during breeding favored alternative genomic regions controlling fw or that these alleles were not retained during later improvement. For INDEL-associated loci, several distinct evolutionary patterns were observed (Fig. 6). For INDEL-associated loci, several distinct evolutionary patterns were observed **(**Fig. 6**).** The clearest domestication signature was detected at chr4_13,734,554 bp, where the INDEL allele associated with increased fw was absent in SP, emerged in SLC, and became nearly fixed in SLL. A distinct evolutionary pattern was observed at chr5_55,361,233 bp, where the fw increasing INDEL allele was absent from both SP and SLC but present exclusively in SLL **(**Fig. 6b**)**. This allele was significantly associated with increased fw and was repeatedly detected across Florida, Georgia, and Valencia. Unlike loci showing progressive enrichment from SP to SLC to SLL, the distribution of this allele suggests a more recent origin or introduction during the crop improvement **(**Fig. 6b**)**. Several INDEL loci exhibited the opposite pattern, where alleles associated with reduced fw were progressively eliminated during domestication and crop improvement. Representative examples include chr7_4,196,768 bp (Ref: GT; Alternative: G) and chr8_58,643,238 bp (Ref: AT; Alternative: A), where the INDEL allele was associated with decreased fw (effects ranging from − 0.14 to − 0.16) and declined in frequency from SP to SLC before becoming rare or absent in cultivated SLL **(**Fig. 6c and d**)**. These patterns suggest that selection favored the reference alleles at these loci, resulting in the gradual removal of unfavorable variants contributing to smaller fruit size. Analysis of SV revealed several distinct evolutionary trajectories underlying fw variation **(**Fig. 7**).** Many SV alleles associated with reduced fw were progressively eliminated during domestication and crop improvement. Representative examples include the SVs at chr2_45,709,317 bp (378-bp deletion), chr4_5,630,658 bp (9,486-bp deletion), chr9_22,330,015 bp (1,040-bp deletion), and chr9_22,320,021 bp (11,886-bp duplication). At these loci, the SV allele was common in SP, declined in frequency in SLC, and was completely absent or nearly absent in cultivated SLL. Consistent with this evolutionary trajectory, accessions carrying the SV allele exhibited significantly lower fw than those carrying the reference allele, indicating that selection during domestication favored the reference haplotype and progressively purged SV alleles associated with reduced fruit size **(**Fig. 7a and b**)**. Unlike these loci, the 390-bp deletion at chr5_45,551,013 bp was associated with increased fw and exhibited a markedly different evolutionary trajectory. The deletion allele was absent in both SP and SLC but present in cultivated SLL accessions, suggesting that it likely arose recently or was selectively enriched during the improvement phase of tomato breeding **(**Fig. 7c and d**)**. Notably, this SV represented the most stable structural variant identified in the study, being significantly associated with fw across all four environments and reaching a maximum significance of –log_10_(P) = 18.85 in Florida. The unique distribution and strong phenotypic effect of this deletion indicate that it may represent a relatively recent improvement allele that contributed to the development of larger-fruited cultivated tomatoes. In contrast to these patterns, the 413-bp deletion at chr12_38,911,139 bp exhibited a distinct evolutionary trajectory **(**Fig. 7c and d**)**. The deletion allele was absent in SP, emerged in SLC, and was significantly associated with increased fw (effect = 0.37; –log_10_(P) = 8.92 in Florida). However, the frequency of this deletion subsequently declined and was ultimately lost in cultivated SLL. This pattern suggests that potentially beneficial alleles present in semi-domesticated germplasm may have been left behind or counter-selected during later stages of crop improvement. As a result, this locus may represent a source of favorable fw variation that is largely absent from modern cultivated tomato germplasm.

### Genomic heritability captured by SNP, INDEL, and SV marker sets

Marker-specific genomic heritability estimates were high and similar across the three variant classes, indicating that SNPs, INDELs, and SVs each captured a large proportion of the genetic signal underlying fw. Bayesian RKHS models estimated genomic heritability values of 0.9668 for SNPs, 0.9629 for INDELs, and 0.9606 for SVs, corresponding to 96.68%, 96.29%, and 96.06% of the modeled variance, respectively (Supplementary Table S3). SNPs captured the largest proportion of genomic variance, but the differences among marker classes were small. These results indicate that the three marker systems captured highly overlapping components of the genetic architecture of fw, consistent with the modest differences in predictive ability observed among marker classes.

### Predictive performance across models and environments

**Fig. 8:**
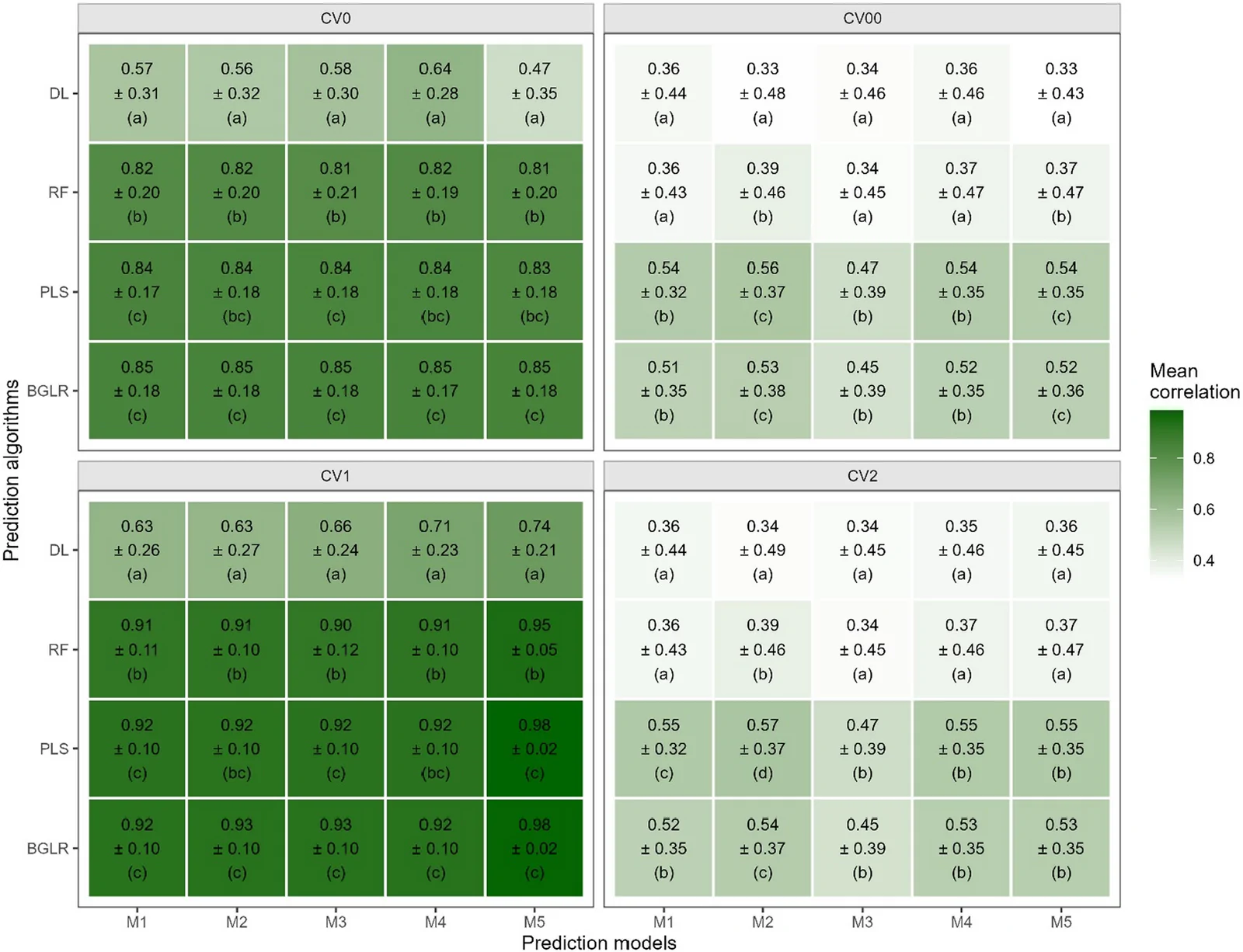
Predictive performance of genomic prediction algorithms across five genomic-source models, four algorithms and four cross-validation strategies. Heatmap cells show the mean prediction accuracy, measured as correlation ± SD, for four prediction algorithms (BGLR, PLS, RF, and DL) across five genomic prediction models: M1 = SNP, M2 = INDEL, M3 = SV, M4 = SNP + INDEL + SV, and M5 = M4 + *G* × *E* interactions. Panels represent the four cross-validation schemes (CV1, CV2, CV0, and CV00). Black boxes indicate the best-performing model–algorithm combination within each cross-validation scheme, except for CV1. Pearson correlation between observed and predicted fruit weight BLUPs. Values within each cell represent the mean prediction accuracy ± standard deviation across 100 random train–test partitions. Letters in parentheses denote Tukey-adjusted groupings obtained from linear mixed-effects models fitted separately for each cross-validation scenario; algorithms sharing the same letter within a prediction model are not significantly different at *P* < 0.05. Color intensity reflects the magnitude of the mean prediction accuracy, with darker green indicating higher predictive performance

The predictive performance of the five genomic prediction models varied substantially across cross-validation (CV) scenarios, reflecting differences in model complexity, genomic information content, and the degree of genetic and environmental novelty. Overall, prediction accuracy declined progressively from CV1 to CV00, consistent with increasing prediction difficulty, yet consistent trends among models were evident (Fig. 8). Overall, prediction accuracy was highest when genotypes and environments were represented in the training data and decreased under scenarios involving untested genotypes and/or unobserved environments. In CV1, where tested genotypes were predicted in observed environments, all models achieved high prediction accuracies except DL. The highest performance was obtained using PLS and BGLR, with maximum correlations reaching 0.98 ± 0.02 for the combined marker model (M5). RF also performed well (0.90–0.95), whereas DL showed substantially lower accuracies (0.63–0.74). In the more challenging CV2 scenario, corresponding to prediction of untested genotypes in observed environments, PLS performance was relatively better across models, with a maximum correlation of 0.57 ± 0.37 for M2 (INDELs). BGLR showed comparable but slightly lower performance (0.45–0.54), whereas RF and DL exhibited markedly lower predictive ability (0.34–0.39 and 0.34–0.36, respectively). For CV0, where tested genotypes were predicted in unobserved environments, BGLR and PLS produced the comparatively highest accuracies once compared with the DL. These results suggest that BGLR is particularly effective for leveraging information from previously evaluated genotypes when extrapolating across environments. The most stringent scenario, CV00, involved prediction of untested genotypes in unobserved environments. Under this setting, PLS and BGLR again provided the highest prediction accuracies achieving 0.52–56 ± 0.35–38 with M2 (INDEL-based model) and M5 (SNP + INDEL + SV + GXE) models. RF and DL showed substantially lower accuracies (< 0.40). Notably, the M2 (INDEL) model was ranked the best-performing models in CV2 (BGLR and PLS), indicating that INDEL variation may capture highly informative signals for predicting fw under conditions relevant to practical breeding programs. Across algorithms, PLS and BGLR consistently outperformed RF and DL, particularly under scenarios involving untested genotypes (CV2 and CV00). Although RF and DL may benefit from further optimization of hyperparameters and larger training populations, their relatively large standard deviations and lower accuracies suggest less stable performance in the present dataset. Increasing the LD threshold increased the number of retained SNPs from 10,976 (*r*^2^ = 0.1) to 218,981 (*r*^2^ = 0.9) (Supplementary Fig. S8a). Marker density had little effect on prediction accuracy under the CV1 and CV0 scenarios, which remained consistently high across all LD thresholds (approximately 0.92 and 0.84–0.85, respectively) (Supplementary Fig. S8b). In contrast, increasing marker density improved prediction accuracy under the more challenging CV2 and CV00 scenarios, with mean correlations increasing from 0.51 to 0.60 for CV2 and from 0.51 to 0.59 for CV00. These results indicate that increasing marker density primarily benefits prediction of untested genotypes across environments, whereas prediction of tested genotypes is largely unaffected by marker density. Additional prediction metrics, including RMSE, MAE, and prediction bias, are summarized in Supplementary Fig. S9 and provide complementary evidence for evaluating model performance beyond correlation-based prediction accuracy.

## Discussion

In this study, we present a comprehensive evaluation of the genetic architecture, evolutionary dynamics, and predictability of tomato fw by jointly integrating SNPs, INDELs, and SVs across multiple environments and domestication stages. By combining multi-environment phenotypic analyses, GWAS, and genomic prediction, we demonstrate that fw is a highly heritable and genetically canalized trait whose underlying variation has been differentially shaped by domestication and improvement. Importantly, our results show that distinct classes of genomic variants contribute unequally to fw variation and its predictability, and that explicitly leveraging this heterogeneity substantially improves both biological insight and predictive performance (Zhou et al. 2022; Li et al. 2023). Our findings complement and extend recent tomato pangenome studies by shifting the emphasis from genome-resource development and SV discovery (Zhou et al. 2022; Li et al. 2023) toward the applied use of multiple variant classes for prediction of a major domestication and breeding trait. Previous graph- and super-pangenome studies demonstrated that SVs contribute substantially to hidden genomic diversity, missing heritability, metabolite variation, flavor-related traits, and wild-species diversity in tomato (Zhou et al. 2022; Li et al. 2023; Shi et al. 2026). In contrast, the present study evaluates SNPs, INDELs, and SVs within a unified multi-environment GWAS and genomic prediction framework for fw. By comparing variant classes across cross-validation scenarios and relating associated alleles to SP, SLC, and SLL, our results provide a trait-focused and breeding-oriented assessment of how different forms of genomic variation contribute to fw prediction across the tomato domestication continuum.

### Genetic control and environmental robustness of fruit weight

Consistent with the variance component analyses, fw was overwhelmingly controlled by genetic effects, with genotype explaining more than 92.1% of total phenotypic variance and only marginal contributions from environment and *G* × *E* interactions. The predominance of genetic variance observed in this study agrees with previous reports showing that fw is among the most heritable traits in tomato and is strongly influenced by major-effect loci selected during domestication and breeding (Pereira et al. 2021). Reaction norm analyses further revealed a progressive reduction in environmental sensitivity from wild SP to cultivated SLL, suggesting that selection during domestication and improvement favored stable fruit size across variable environments. Furthermore, domesticated tomato groups (particularly SLL and semi-domesticated SLC) maintain relatively stable fw across diverse environments, whereas the wild progenitor SP exhibits greater environmental sensitivity and variation in fw expression. These findings align with previous reports that fw is among the most genetically buffered yield components in tomato and they provide a phenotypic foundation for the strong and reproducible GWAS, and genomic prediction signals detected in this study (Lin et al. 2014; Barrantes et al. 2016; Pereira et al. 2021).

### GWAS reveals stable and environment-specific fruit weight loci

GWAS across four independent environments identified fw-associated loci for all three variant classes, distributed across all 12 chromosomes. Several loci exhibited remarkable environmental stability, particularly the SNP region on chr 2 (45.66–50.41 Mb), the INDEL at chr5_55,361,233 bp, and the SV at chr5_45,551,013 bp, which were repeatedly detected across multiple environments. These repeatedly detected signals are consistent with the high heritability and limited genotype-by-environment interactions observed at the phenotypic level, identifying these regions as core components of the genetic architecture of fw (Alonge et al. 2020; Pereira et al. 2021). Among the GWAS loci identified, several QTL co-localized with previously characterized fw genes that were major targets of human selection during the two-step evolution from wild SP to modern big-fruited varieties (SLL), while others appeared as stable loci across environments (Lin et al. 2014; Pereira et al. 2021). The SNP at chr2_50,414,767 bp was located approximately 121 kb downstream of *fw2.2/CNR* (*Solyc02g090730*) (Frary et al. 2000), while the SV at chr3_59,206,418 bp was located approximately 11 kb upstream of *fw3.2/SlKLUH* (*Solyc03g114940*) (Chakrabarti et al. 2013; Alonge et al. 2020). In addition, a cluster of significant SNPs on chr 11 (52.95–53.30 Mb) coincided with the genomic region harboring *fw11.3/CSR* (*Cell Size Regulator*, *Solyc11g071940*) and *fas/CLAVATA3* (Mu et al. 2017; Chu et al. 2019). A stable chr 2 association spanning 45.66–45.78 Mb co-localized with the classical *lc/WUSCHEL* fruit size locus (Chu et al. 2019). Both the SNP at chr2_45,663,367 bp and the SV at chr2_45,709,317 bp were located within approximately 120 kb of *lc/WUSCHEL* (*Solyc02g083950*), a major domestication gene controlling locule number and fruit size in tomato (Chu et al. 2019). The repeated detection of this region across multiple environments suggests that allelic variation linked to the *lc* locus continues to contribute to natural fw variation within the Varitome collection. The identification of SNP, INDEL, and SV associations near both *lc/WUSCHEL* and *fas/CLAVATA3* is particularly significant because these loci were independently selected during tomato domestication and are known to increase fw through enhanced locule number and enlarged meristems (Rodríguez et al. 2011; Blanca et al. 2015, 2022). The persistence of significant associations surrounding both the *lc/WUSCHEL* and *fas/CLAVATA3* regions suggests that these major domestication loci continue to contribute to fw variation in modern tomato germplasm (Chu et al. 2019). Furthermore, the co-localization of SNP, INDEL, and SV associations with *lc, fas, fw2.2, fw3.2* and *fw11.3* provides strong biological support for the detected signals and highlights the value of integrating multiple variant classes to improve the resolution and robustness of association mapping. In addition to these cloned genes, our analysis consistently detected other environment-stable QTLs across all tested environments. These repeatedly significant regions likely represent robust, additive loci contributing to fw stability under variable conditions and are promising targets for functional validation and marker-assisted selection. These findings confirm that multiple variant classes converge around key developmental regulators and support their combined use for improving the resolution of association mapping. Although most LD intervals were well captured by the 400-kb window, the chr4_13,734,554 and chr11_31,865,292 INDEL loci showed extended LD and were therefore reanalyzed using an expanded 1-Mb interval. These broader LD patterns are consistent with low-recombination or pericentromeric genomic regions in tomato (Tomato Genome Consortium 2012), where association signals may represent extended haplotypes rather than narrowly resolved causal variants. Therefore, candidate intervals in these regions should be interpreted cautiously, and additional fine-mapping populations or recombinant screening will be required to resolve the causal genes or variants. Altogether, further validation through progeny testing, expression profiling, or CRISPR-based mutagenesis will be essential to confirm causality and identify functional variants underpinning the QTL identified in our study.

### Evolutionary scenarios across domestication stages

Our GWAS results revealed distinct evolutionary trajectories shaping fw alleles across domestication stages. Several favorable alleles showed the expected progressive increase in frequency from SP to SLC and ultimately fixation in SLL, consistent with selection for larger fruit during domestication (Blanca et al. 2015, 2022; Alonge et al. 2020; Singh and van der Knaap 2022). Particularly striking was the chr 2 region spanning 45.66–50.41 Mb, where multiple SNPs displayed nearly identical allele-frequency trajectories and co-localized with *fw2.2/CNR* and *lc/WUSCHEL*, suggesting that this region has been a major target of selection throughout tomato domestication. Similar patterns, where beneficial alleles for stress adaptation were left behind as selection favored larger fruits and higher yield, have been reported in tomato and other crops (Doebley et al. 2006; Meyer and Purugganan 2013; Lin et al. 2014; Razifard et al. 2020; Zhou et al. 2022). In contrast, the stable chr 5 loci displayed a markedly different pattern. Both the INDEL at chr5_55,361,233 bp and the SV at chr5_45,551,013 bp were associated with increased fw and exhibited some of the strongest and most consistent effects detected in this study. However, unlike classical domestication loci, the favorable alleles were absent from both SP and SLC and occurred almost exclusively in SLL germplasm in Varitome collection. This distribution suggests that these alleles likely arose or were introduced after the domestication transition and became rapidly fixed during modern crop improvement, similar to the improvement-associated fruit shape gene *fs8.1* (Zhu et al. 2023). Their strong positive effects on fw, combined with their near fixation in cultivated germplasm, indicate that chr 5 represents an important improvement stage target distinct from the major domestication loci such as *fw2.2* and *fw3.2*. Some INDELs absent in SP but present in SLC and SLL likely represent introgressed variants that were selected for increased fruit size, such as those located on chr9 (chr9_56,914,628) and Chr10 (chr10_62,579,411). Conversely, in the SV dataset, certain large-fruit alleles were lost during the SLC–SLL transition, for example at chr12 (chr12_38,911,139 bp), which may indicate trade-offs among fruit size, yield stability, and stress tolerance (Razifard et al. 2020; Singh and van der Knaap 2022). Together, these findings demonstrate how domestication selectively filtered allelic variation in both directions, reinforcing the polygenic and context-dependent nature of fw regulation in tomato.

### Variant-type contributions to genomic prediction

Our findings highlight how evolutionary processes associated with tomato domestication and improvement have shaped not only allele frequencies but also the relative informativeness of distinct variant types. Across genomic prediction analyses, prediction performance was influenced more strongly by cross-validation scenario and prediction algorithm than by marker class alone. INDEL-based models appeared among the best-performing approaches and achieved the highest prediction accuracies under the challenging prediction scenarios (CV2) using BGLR and PLS algorithms. However, differences among marker classes were generally modest, suggesting that SNPs, INDELs, and SVs capture overlapping components of the genetic architecture underlying fw (Alonge et al. 2020; Zhou et al. 2022; Li et al. 2023). Therefore, the advantage of INDEL-based prediction should be interpreted as a trend observed in this dataset rather than as a universal superiority of INDELs across traits or populations. Nevertheless, the combined SNP + INDEL + SV marker panel did not consistently outperform the INDEL marker set, suggesting that the three marker classes captured largely overlapping genetic information because many variants tagged the same genomic regions through linkage disequilibrium. Thus, the practical value of the multi-variant framework should not be interpreted primarily as a large improvement in prediction accuracy. Instead, its main strength lies in improving biological interpretation, identifying candidate loci, and clarifying which classes of genomic variation capture meaningful fw-associated signals. This interpretation is supported by the similar genomic heritability estimates obtained for SNPs, INDELs, and SVs. Furthermore, the relatively superior predictive performance of INDEL-based models suggests that small insertions and deletions capture a larger proportion of functionally relevant genetic variation underlying fw. This is biologically plausible, as INDELs are more likely than SNPs to directly affect gene function by disrupting coding sequences, altering protein structure, or modifying regulatory elements such as promoters and untranslated regions (Shomura et al. 2008; Studer et al. 2011; Taylor et al. 2019; Alonge et al. 2020). Consequently, despite being less abundant than SNPs, INDELs may capture a larger proportion of causative variation underlying fw. This interpretation is consistent with previous studies showing that prediction accuracy does not necessarily increase with marker number alone and that functionally informative or multiallelic variants can outperform substantially larger SNP datasets (Li et al. 2018; Luo et al. 2021; Heinrich et al. 2023; Mota et al. 2025). Furthermore, our marker density analysis using LD pruning showed also that marker number alone was not the limiting factor. Marker density had little effect on prediction accuracy under the less stringent CV1 and CV0 scenarios but resulted in modest improvements under the more challenging CV2 and CV00 scenarios. Thus, our results reinforce the notion that optimizing marker quality and biological relevance may be more important than maximizing marker quantity for genomic prediction. Although integrating multiple marker classes can capture complementary sources of genetic variation, adding additional variant classes does not necessarily improve prediction if those markers contribute redundant or weakly informative signals. For a highly heritable trait such as fw, where several major domestication-related loci contribute strongly to phenotypic variation, INDELs may already tag much of the relevant functional variation. Thus, combined models may increase model complexity without a proportional increase in informative genetic signal. Similarly, SVs, despite of being less abundant than SNPs and INDELs, also contributed to fw variation. Several SV-associated loci co-localized with known fw QTLs, supporting their functional importance in tomato (Maron et al. 2013; Alonge et al. 2020; Zhou et al. 2022). SVs can induce large phenotypic effects through mechanisms such as gene dosage changes, gene disruption, or chromosomal rearrangements, which may directly impact developmental pathways controlling fruit growth (Alonge et al. 2020; Garg et al. 2025). Their comparatively lower predictive accuracy likely reflects limited allele representation and short-read detection limits, yet integrating SVs with SNPs and INDELs yielded modest, consistent gains matching evidence that multi-variant genomic frameworks can capture additional variance and help recover missing heritability (Maron et al. 2013; Della Coletta et al. 2021; Zhou et al. 2022). These variants play disproportionate roles in phenotypic differentiation and trait heritability, underscoring the importance of integrating multiple types of genomic variation rather than relying exclusively on SNPs (Hämälä et al. 2021; Zhou et al. 2022; Wang et al. 2025b). Therefore, their inclusion remains important for candidate-locus discovery and biological interpretation, even when they do not produce consistently higher prediction accuracy than SNPs or combined-marker models. Together, these results support the use of multi-variant genomic frameworks for integrating predictive modeling with biological interpretation. Rather than identifying a universally superior marker class, our results show that SNPs, INDELs, and SVs provide complementary perspectives on fw architecture, domestication history, and candidate-locus prioritization. Additionally, the practical value of the multi-variant framework should not be interpreted solely as a gain in prediction accuracy, but also as an improved understanding of which classes of genomic variation capture biologically meaningful fw variation. Marker-density differences among SNPs, INDELs, and SVs may also influence prediction performance. Future studies using independent validation populations, functional assays, and density-matched marker sets will be useful for evaluating the robustness of variant-class-specific prediction trends.

### Predictive performance of genomic prediction algorithms

Prediction accuracy was strongly influenced by the choice of prediction algorithm and validation scenario (Ortiz et al. 2023; Montesinos-López et al. 2025). Across nearly all cross-validation schemes, PLS and BGLR consistently outperformed RF and DL, particularly under the more challenging CV2 and CV00 scenarios involving untested genotypes. The strong performance of PLS is consistent with previous studies showing that dimension-reduction approaches can efficiently capture latent genetic signals and improve prediction accuracy when extrapolating to new environments or breeding cycles (Wold et al. 2001; Rosipal and Krämer 2006; Boulesteix and Strimmer 2007; Montesinos-López et al. 2021, 2022). In particular, PLS has been shown to outperform conventional genomic prediction methods, including GBLUP, across multiple crops and multi-environment datasets when predicting future seasons or novel environments (Montesinos-López et al. 2022; Ortiz et al. 2023). Furthermore, PLS was among the top-performing methods for maize grain yield prediction using integrated genomic, metabolomic, and image-derived phenotypes, highlighting its ability to efficiently extract informative signals from highly correlated predictor variables (Wu et al. 2024) Likewise, the strong performance of BGLR likely reflects its ability to model additive marker effects and realized genomic relationships among individuals, which remain highly informative for traits governed primarily by additive genetic variation (Pérez and de los Campos 2014; Chafai et al. 2023). The relatively small differences observed among prediction models are also consistent with previous studies showing that no single algorithm consistently outperforms all others across datasets and traits. Instead, predictive performance often depends on the underlying genetic architecture, data structure, and prediction objective (Chafai et al. 2023; Wu et al. 2024). In contrast, the comparatively lower performance of RF and DL is consistent with several reviews and benchmarking studies reporting that more complex machine-learning approaches do not consistently outperform conventional genomic prediction methods, particularly when training populations are moderate and trait architecture is largely additive (Chafai et al. 2023).

### Implications of limited G × E for genomic prediction

Despite the wide environmental range represented by the study sites, *G* × *E* interactions explained only a small portion of the phenotypic variance. This was reflected both in variance partitioning analyses and in the relatively small gains achieved by explicitly modeling *G* × *E* interactions (M5) in genomic prediction. These results indicate that fw is largely governed by stable, additive genetic effects that are robust across moderate environmental contrasts, consistent with previous reports in tomato and other crops (Lin et al. 2014; Barrantes et al. 2016; Pereira et al. 2021). Together with the strong cross-environment correlations observed among environments, the high prediction accuracies achieved under the most stringent CV2 and CV00 scenarios support the notion that core genetic determinants of fw can be reliably captured and transferred across environments and genetic backgrounds.

Taken together, our results demonstrate that fw is primarily controlled by stable additive genetic effects that can be accurately predicted across environments and genetic backgrounds. While INDEL-based models frequently achieved the highest accuracies under challenging prediction scenarios, differences among marker classes were generally smaller than differences among prediction algorithms. PLS and BGLR consistently provided the most reliable predictions, whereas RF and DL offered little advantage despite their greater complexity. These findings suggest that successful genomic prediction depends not only on marker density but also on selecting variant classes and analytical approaches that efficiently capture biologically meaningful genetic variation. Integrating SNPs, INDELs, and SVs therefore provides a valuable framework for simultaneously improving biological interpretation, identifying functional loci, and understanding the evolutionary processes that shaped fw during tomato domestication and improvement.

## Supplementary Information

Below is the link to the electronic supplementary material.

Supplementary file1 (DOCX 6173 KB)

Supplementary file2 (XLSX 33 KB)

## Data Availability

All scripts and input files required to reproduce the multi-environment genomic prediction analyses are publicly available at the GitHub repository: https://github.com/yasin-topcu/Genomic-prediction-with-SNPs-INDELs-and-SVs. The repository contains R scripts implementing the genomic prediction analyses using BGLR, PLS, RF, and DL, including the calculation of prediction accuracy, RMSE, MAE, and prediction bias. It also provides the genomic relationship matrices for SNPs (GRM.GAPIT.csv), INDELs (GRM_indel.csv), and structural variants (GRM_sv.csv), the phenotypic datasets (Blues.Pheno.corrected.csv and pedigree_env_blups_fixedG.csv), the predefined 100 replicated 70/30 training–testing partitions (geno_70_30_splits_100x.RData), the corresponding prediction results (Split_results_100x.RData), the structural variant HapMap file (Hapmap.SVs.hmp.zip), and the scripts required for LD pruning. A detailed README file accompanies the repository and describes the purpose, format, required structure, and expected outputs of each file, enabling full reproduction of the genomic prediction analyses presented in this study.

## References

[CR1] Alonge M, Wang X, Benoit M, Soyk S, Pereira L, Zhang L, Suresh H, Ramakrishnan S, Maumus F, Ciren D et al (2020) Major impacts of widespread structural variation on gene expression and crop improvement in tomato. Cell. 10.1016/j.cell.2020.05.021 32553272 PMC7354227

[CR2] Barrantes W, López-Casado G, García-Martínez S, Alonso A, Rubio F, Ruiz JJ, Fernández-Muñoz R, Granell A, Monforte AJ (2016) Exploring new alleles involved in tomato fruit quality in an introgression line library of *Solanum**pimpinellifolium*. Front Plant Sci. 10.3389/fpls.2016.01172 27582742 PMC4987366

[CR3] Bates D, Mächler M, Bolker B, Walker S (2015) Fitting linear mixed-effects models using lme4. J Stat Softw. 10.18637/jss.v067.i01

[CR4] Blanca J, Montero-Pau J, Sauvage C, Bauchet G, Illa E, Díez MJ, Francis D, Causse M, van der Knaap E, Cañizares J (2015) Genomic variation in tomato, from wild ancestors to contemporary breeding accessions. BMC Genomics. 10.1186/s12864-015-1444-1 25880392 PMC4404671

[CR5] Blanca J, Sanchez-Matarredona D, Ziarsolo P, Montero-Pau J, van der Knaap E, Díez MJ, Cañizares J (2022) Haplotype analyses reveal novel insights into tomato history and domestication driven by long-distance migrations and latitudinal adaptations. Hortic Res. 10.1093/hr/uhac030 35184177 PMC8976693

[CR6] Bolger AM, Lohse M, Usadel B (2014) Trimmomatic: a flexible trimmer for Illumina sequence data. Bioinformatics. 10.1093/bioinformatics/btu170 24695404 PMC4103590

[CR7] Boulesteix A-L, Strimmer K (2007) Partial least squares: a versatile tool for the analysis of high-dimensional genomic data. Brief Bioinform. 10.1093/bib/bbl016 16772269

[CR8] Bradbury PJ, Zhang Z, Kroon DE, Casstevens TM, Ramdoss Y, Buckler ES (2007) TASSEL: software for association mapping of complex traits in diverse samples. Bioinformatics. 10.1093/bioinformatics/btm308 17586829

[CR9] Cappetta E, Andolfo G, Di Matteo A, Barone A, Frusciante L, Ercolano MR (2020) Accelerating tomato breeding by exploiting genomic selection approaches. Plants (Basel). 10.3390/plants9091236 32962095 PMC7569914

[CR10] Chafai N, Hayah I, Houaga I, Badaoui B (2023) A review of machine learning models applied to genomic prediction in animal breeding. Front Genet. 10.3389/fgene.2023.1150596 37745853 PMC10516561

[CR11] Chakrabarti M, Zhang N, Sauvage C, Muños S, Blanca J, Cañizares J, Diez MJ, Schneider R, Mazourek M, McClead J et al (2013) A cytochrome P450 regulates a domestication trait in cultivated tomato. Proc Natl Acad Sci U S A. 10.1073/pnas.1307313110 24082112 PMC3801035

[CR12] Chu Y-H, Jang J-C, Huang Z, van der Knaap E (2019) Tomato locule number and fruit size controlled by natural alleles of lc and fas. Plant Dir. 10.1002/pld3.142 PMC6607973 31312784

[CR13] Cui X, Liu Y, Sun M, Zhao Q, Huang Y, Zhang J, Yao Q, Yin H, Zhang H, Mo F et al (2025) The nature of complex structural variations in tomatoes. Hortic Res. 10.1093/hr/uhaf107 40406505 PMC12096311

[CR14] Della Coletta R, Qiu Y, Ou S, Hufford MB, Hirsch CN (2021) How the pan-genome is changing crop genomics and improvement. Genome Biol. 10.1186/s13059-020-02224-8 33397434 PMC7780660

[CR15] Delourme R, Falentin C, Fomeju BF, Boillot M, Lassalle G, André I, Duarte J, Gauthier V, Lucante N, Marty A et al (2013) High-density SNP-based genetic map development and linkage disequilibrium assessment in *Brassica napus* L. BMC Genomics. 10.1186/1471-2164-14-120 23432809 PMC3600037

[CR16] Doebley JF, Gaut BS, Smith BD (2006) The molecular genetics of crop domestication. Cell. 10.1016/j.cell.2006.12.006 17190597

[CR17] Frary A, Nesbitt TC, Frary A, Grandillo S, Knaap E, Cong B, Liu J, Meller J, Elber R, Alpert KB et al (2000) Fw2.2: a quantitative trait locus key to the evolution of tomato fruit size. Science. 10.1126/science.289.5476.85 10884229

[CR18] Garg V, Barmukh R, Huang Y, Chitikineni A, Hobson K, Yang B, Jia Y, Bi S, Kaur S, Asif MA et al (2025) An Australian chickpea pan-genome provides insights into genome organization and offers opportunities for enhancing drought adaptation for crop improvement. Plant Biotechnol J. 10.1111/pbi.70192 40534107 PMC12392939

[CR19] Hämälä T, Wafula EK, Guiltinan MJ, Ralph PE, dePamphilis CW, Tiffin P (2021) Genomic structural variants constrain and facilitate adaptation in natural populations of *Theobroma cacao*, the chocolate tree. Proc Natl Acad Sci U S A. 10.1073/pnas.2102914118 34408075 PMC8536383

[CR20] Heinrich F, Lange TM, Kircher M, Ramzan F, Schmitt AO, Gültas M (2023) Exploring the potential of incremental feature selection to improve genomic prediction accuracy. Genet Sel Evol. 10.1186/s12711-023-00853-8 37946104 PMC10634161

[CR21] Heslot N, Yang H-P, Sorrells ME, Jannink J-L (2012) Genomic selection in plant breeding: A comparison of models. Crop Sci. 10.2135/cropsci2011.06.0297

[CR22] Kayikci HC, Aydin S, Adak A, Dogan A, Sapkota M, Feng Q, Topcu Y (2025) Association mapping of tomato fruit quality for weight, firmness, brix, and color using GWAS. BMC Plant Biol. 10.1186/s12870-025-07838-3 41331747 PMC12781814

[CR23] Li X, Han Y, Wei Y, Acharya A, Farmer AD, Ho J, Monteros MJ, Brummer EC (2014) Development of an alfalfa SNP array and its use to evaluate patterns of population structure and linkage disequilibrium. PLoS ONE. 10.1371/journal.pone.0084329 24416217 PMC3887001

[CR24] Li B, Zhang N, Wang Y-G, George AW, Reverter A, Li Y (2018) Genomic prediction of breeding values using a subset of SNPs identified by three machine learning methods. Front Genet. 10.3389/fgene.2018.00237 30023001 PMC6039760

[CR25] Li W, Liu J, Zhang H, Liu Z, Wang Y, Xing L, He Q, Du H (2022) Plant pan-genomics: recent advances, new challenges, and roads ahead. J Genet Genomics. 10.1016/j.jgg.2022.06.004 35750315

[CR26] Li N, He Q, Wang J, Wang B, Zhao J, Huang S, Yang T, Tang Y, Yang S, Aisimutuola P et al (2023) Super-pangenome analyses highlight genomic diversity and structural variation across wild and cultivated tomato species. Nat Genet. 10.1038/s41588-023-01340-y 37024581 PMC10181942

[CR27] Lin T, Zhu G, Zhang J, Xu X, Yu Q, Zheng Z, Zhang Z, Lun Y, Li S, Wang X et al (2014) Genomic analyses provide insights into the history of tomato breeding. Nat Genet. 10.1038/ng.3117 25305757

[CR28] Luo Z, Yu Y, Xiang J, Li F (2021) Genomic selection using a subset of SNPs identified by genome-wide association analysis for disease resistance traits in aquaculture species. Aquaculture. 10.1016/j.aquaculture.2021.736620

[CR29] Ma W, Qiu Z, Song J, Li J, Cheng Q, Zhai J, Ma C (2018) A deep convolutional neural network approach for predicting phenotypes from genotypes. Planta. 10.1007/s00425-018-2976-9 30101399

[CR30] Maron LG, Guimarães CT, Kirst M, Albert PS, Birchler JA, Bradbury PJ, Buckler ES, Coluccio AE, Danilova TV, Kudrna D et al (2013) Aluminum tolerance in maize is associated with higher *MATE1* gene copy number. Proc Natl Acad Sci U S A. 10.1073/pnas.1220766110 23479633 PMC3612656

[CR31] Mata-Nicolás E, Montero-Pau J, Gimeno-Paez E, Garcia-Carpintero V, Ziarsolo P, Menda N, Mueller LA, Blanca J, Cañizares J, van der Knaap E et al (2020) Exploiting the diversity of tomato: the development of a phenotypically and genetically detailed germplasm collection. Hortic Res. 10.1038/s41438-020-0291-7 32377357 PMC7192925

[CR32] Meuwissen THE, Hayes BJ, Goddard ME (2001) Prediction of total genetic value using genome-wide dense marker maps. Genetics. 10.1093/genetics/157.4.1819 11290733 PMC1461589

[CR33] Meyer RS, Purugganan MD (2013) Evolution of crop species: genetics of domestication and diversification. Nat Rev Genet. 10.1038/nrg3605 24240513

[CR34] Montesinos López OA, Mosqueda González BA, Palafox González A, Montesinos López A, Crossa J (2022) A general-purpose machine learning R library for sparse kernel methods with an application for genome-based prediction. Front Genet. 10.3389/fgene.2022.887643 35719365 PMC9205295

[CR35] Montesinos López OA, Mosqueda González BA, Montesinos López A, Crossa J (2023) Statistical machine-learning methods for genomic prediction using the SKM library. Genes. 10.3390/genes14051003 37239363 PMC10218433

[CR36] Montesinos-López A, Montesinos-López OA, Gianola D, Crossa J, Hernández-Suárez CM (2018) Multi-environment genomic prediction of plant traits using deep learners with dense architecture. G3 (Bethesda). 10.1534/g3.118.200740 30291107 PMC6288841

[CR37] Montesinos-López OA, Montesinos-López A, Pérez-Rodríguez P, Barrón-López JA, Martini JWR, Fajardo-Flores SB, Gaytan-Lugo LS, Santana-Mancilla PC, Crossa J (2021) A review of deep learning applications for genomic selection. BMC Genomics. 10.1186/s12864-020-07319-x 33407114 PMC7789712

[CR38] Montesinos-López OA, Montesinos-López A, Kismiantini R-G, Gardner K, Lillemo M, Fritsche-Neto R, Crossa J (2022) Partial least squares enhances genomic prediction of new environments. Front Genet. 10.3389/fgene.2022.920689 36313422 PMC9608852

[CR39] Montesinos-López A, Crespo-Herrera L, Dreisigacker S, Gerard G, Vitale P, Saint Pierre C, Govindan V, Tarekegn ZT, Flores MC, Pérez-Rodríguez P et al (2024) Deep learning methods improve genomic prediction of wheat breeding. Front Plant Sci. 10.3389/fpls.2024.1324090 38504889 PMC10949530

[CR40] Montesinos-López A, Montesinos-López OA, Ramos-Pulido S, Mosqueda-González BA, Guerrero-Arroyo EA, Crossa J, Ortiz R (2025) Artificial intelligence meets genomic selection: comparing deep learning and GBLUP across diverse plant datasets. Front Genet. 10.3389/fgene.2025.1568705 40364946 PMC12069277

[CR41] Mota LFM, Arikawa LM, Valente JPS, Fonseca LFS, Mercadante MEZ, Cyrillo JNSG, Oliveira HN, Albuquerque LG (2025) Variable selection strategies for genomic prediction of growth and carcass related traits in experimental Nellore cattle herds under different selection criteria. Sci Rep. 10.1038/s41598-025-06949-z 40594941 PMC12218294

[CR42] Mu Q, Huang Z, Chakrabarti M, Illa-Berenguer E, Liu X, Wang Y, Ramos A, van der Knaap E (2017) Fruit weight is controlled by *Cell Size Regulator* encoding a novel protein that is expressed in maturing tomato fruits. PLoS Genet. 10.1371/journal.pgen.1006930 28817560 PMC5560543

[CR43] Muños S, Ranc N, Botton E, Bérard A, Rolland S, Duffé P, Carretero Y, Le Paslier M-C, Delalande C, Bouzayen M et al (2011) Increase in tomato locule number is controlled by two single-nucleotide polymorphisms located near *WUSCHEL*. Plant Physiol. 10.1104/pp.111.173997 21673133 PMC3149950

[CR44] Nelson MN, Książkiewicz M, Rychel S, Besharat N, Taylor CM, Wyrwa K, Jost R, Erskine W, Cowling WA, Berger JD et al (2017) The loss of vernalization requirement in narrow-leafed lupin is associated with a deletion in the promoter and de-repressed expression of a *Flowering Locus T (FT)* homologue. New Phytol. 10.1111/nph.14094 27418400

[CR45] Ortiz R, Crossa J, Vargas M, Izquierdo J (2007) Studying the effect of environmental variables on the genotype × environment interaction of tomato. Euphytica. 10.1007/s10681-006-9248-7

[CR46] Ortiz R, Reslow F, Montesinos-López A, Huicho J, Pérez-Rodríguez P, Montesinos-López OA, Crossa J (2023) Partial least squares enhance multi-trait genomic prediction of potato cultivars in new environments. Sci Rep. 10.1038/s41598-023-37169-y 37336933 PMC10279678

[CR47] Pereira L, Zhang L, Sapkota M, Ramos A, Razifard H, Caicedo AL, van der Knaap E (2021) Unraveling the genetics of tomato fruit weight during crop domestication and diversification. Theor Appl Genet. 10.1007/s00122-021-03902-2 34283260 PMC8440300

[CR48] Pérez P, de los Campos G (2014) Genome-wide regression and prediction with the BGLR statistical package. Genetics. 10.1534/genetics.114.164442 25009151 PMC4196607

[CR49] Razifard H, Ramos A, Della Valle AL, Bodary C, Goetz E, Manser EJ, Li X, Zhang L, Visa S, Tieman D et al (2020) Genomic evidence for complex domestication history of the cultivated tomato in Latin America. Mol Biol Evol. 10.1093/molbev/msz297 31912142 PMC7086179

[CR50] Rodríguez GR, Muños S, Anderson C, Sim S-C, Michel A, Causse M, Gardener BBM, Francis D, van der Knaap E (2011) Distribution of *SUN*, *OVATE*, *LC*, and *FAS* in the tomato germplasm and the relationship to fruit shape diversity. Plant Physiol. 10.1104/pp.110.167577 21441384 PMC3091046

[CR51] Rosipal R, Krämer N (2006) Overview and recent advances in partial least squares. In: Saunders C, Grobelnik M, Gunn S, Shawe-Taylor J (eds) Subspace, latent structure and feature selection. Springer Berlin Heidelberg, Berlin, Heidelberg, pp 34–51

[CR52] Sapkota M, Pereira L, Wang Y, Zhang L, Topcu Y, Tieman D, van der Knaap E (2023) Structural variation underlies functional diversity at methyl salicylate loci in tomato. PLoS Genet. 10.1371/journal.pgen.1010751 37141297 PMC10187894

[CR53] Shi C, Chen S, Wang J, Chen W, Sun C, Guo Q, Liao S, Wang H, Mu Y, Shu X et al (2026) A tomato telomere-to-telomere super-pangenome empowers stress resilience breeding. Nat Genet. 10.1038/s41588-026-02508-y 41708786

[CR54] Shin J-H, Blay S, McNeney B, Graham J (2006) LDheatmap: an R function for graphical display of pairwise linkage disequilibria between single nucleotide polymorphisms. J Stat Softw. 10.18637/jss.v016.c03

[CR55] Shomura A, Izawa T, Ebana K, Ebitani T, Kanegae H, Konishi S, Yano M (2008) Deletion in a gene associated with grain size increased yields during rice domestication. Nat Genet. 10.1038/ng.169 18604208

[CR56] Singh J, van der Knaap E (2022) Unintended consequences of plant domestication. Plant Cell Physiol. 10.1093/pcp/pcac083 35715986

[CR57] Studer A, Zhao Q, Ross-Ibarra J, Doebley J (2011) Identification of a functional transposon insertion in the maize domestication gene *tb1*. Nat Genet. 10.1038/ng.942 21946354 PMC3686474

[CR58] Tanksley SD (2004) The genetic, developmental, and molecular bases of fruit size and shape variation in tomato. Plant Cell. 10.1105/tpc.018119 15131251 PMC2643388

[CR59] Taylor CM, Kamphuis LG, Zhang W, Garg G, Berger JD, Mousavi-Derazmahalleh M, Bayer PE, Edwards D, Singh KB, Cowling WA et al (2019) INDEL variation in the regulatory region of the major flowering time gene *LanFTc1* is associated with vernalization response and flowering time in narrow-leafed lupin (*Lupinus angustifolius* L.). Plant Cell Environ. 10.1111/pce.13320 29677403 PMC7379684

[CR60] Tomato Genome Consortium (2012) The tomato genome sequence provides insights into fleshy fruit evolution. Nature. 10.1038/nature11119 PMC3378239 22660326

[CR61] Tong H, Nikoloski Z (2021) Machine learning approaches for crop improvement: leveraging phenotypic and genotypic big data. J Plant Physiol. 10.1016/j.jplph.2020.153354 33385619

[CR62] Topcu Y, Yildiz K, Kayikci HC, Aydin S, Feng Q, Sapkota M (2025) Deciphering resistance to Tomato brown rugose fruit virus (ToBRFV) using genome-wide association studies. Sci Hortic. 10.1016/j.scienta.2025.113968

[CR63] van der Knaap E, Chakrabarti M, Chu YH, Clevenger JP, Illa-Berenguer E, Huang Z, Keyhaninejad N, Mu Q, Sun L, Wang Y et al (2014) What lies beyond the eye: the molecular mechanisms regulating tomato fruit weight and shape. Front Plant Sci. 10.3389/fpls.2014.00227 24904622 PMC4034497

[CR64] Vos PG, Paulo MJ, Voorrips RE, Visser RG, van Eck HJ, van Eeuwijk FA (2017) Evaluation of LD decay and various LD-decay estimators in simulated and SNP-array data of tetraploid potato. Theor Appl Genet. 10.1007/s00122-016-2798-8 27699464 PMC5214954

[CR65] Wang J, Zhang Z (2021) GAPIT version 3: boosting power and accuracy for genomic association and prediction. Genomics Proteomics Bioinform. 10.1016/j.gpb.2021.08.005 PMC9121400 34492338

[CR66] Wang K, Abid MA, Rasheed A, Crossa J, Hearne S, Li H (2023) DNNGP, a deep neural network-based method for genomic prediction using multi-omics data in plants. Mol Plant. 10.1016/j.molp.2022.11.004 36366781

[CR67] Wang H, Yan S, Wang W, Chen Y, Hong J, He Q, Diao X, Lin Y, Chen Y, Cao Y et al (2025a) Cropformer: an interpretable deep learning framework for crop genomic prediction. Plant Commun. 10.1016/j.xplc.2024.101223 39690739 PMC11956090

[CR68] Wang X, Liu C, Sun X, Sun G, Zong C, Qi Y, Bai Y, Li W, Kong F, Li H et al (2025b) How structural variations influence crop improvement. Int J Mol Sci. 10.3390/ijms26146635 40724888 PMC12295661

[CR69] Wickham H, Chang W, Wickham MH (2016) ggplot2: create elegant data visualisations using the grammar of graphic. Springer-Verlag, New York

[CR70] Wold S, Sjöström M, Eriksson L (2001) PLS-regression: a basic tool of chemometrics. Chemometr Intell Lab Syst. 10.1016/S0169-7439(01)00155-1

[CR71] Wu C, Luo J, Xiao Y (2024) Multi-omics assists genomic prediction of maize yield with machine learning approaches. Mol Breed. 10.1007/s11032-024-01454-z 38343399 PMC10853138

[CR72] Xu C, Liberatore KL, MacAlister CA, Huang Z, Chu Y-H, Jiang K, Brooks C, Ogawa-Ohnishi M, Xiong G, Pauly M et al (2015) A cascade of arabinosyltransferases controls shoot meristem size in tomato. Nat Genet. 10.1038/ng.3309 26005869

[CR73] Xu Z, Li Q, Marchionni L, Wang K (2023) PhenoSV: interpretable phenotype-aware model for the prioritization of genes affected by structural variants. Nat Commun. 10.1038/s41467-023-43651-y 38016949 PMC10684511

[CR74] Zhao J, Sauvage C, Zhao J, Bitton F, Bauchet G, Liu D, Huang S, Tieman DM, Klee HJ, Causse M (2019) Meta-analysis of genome-wide association studies provides insights into genetic control of tomato flavor. Nat Commun. 10.1038/s41467-019-09462-w 30948717 PMC6449550

[CR75] Zheng X, Levine D, Shen J, Gogarten SM, Laurie C, Weir BS (2012) A high-performance computing toolset for relatedness and principal component analysis of SNP data. Bioinformatics. 10.1093/bioinformatics/bts606 23060615 PMC3519454

[CR76] Zhou Y, Zhang Z, Bao Z, Li H, Lyu Y, Zan Y, Wu Y, Cheng L, Fang Y, Wu K et al (2022) Graph pangenome captures missing heritability and empowers tomato breeding. Nature. 10.1038/s41586-022-04808-9 35676474 PMC9200638

[CR77] Zhu Q, Deng L, Chen J, Rodríguez GR, Sun C, Chang Z, Yang T, Zhai H, Jiang H, Topcu Y et al (2023) Redesigning the tomato fruit shape for mechanized production. Nat Plants. 10.1038/s41477-023-01522-w 37723204

