# Supplementary material for: Multi-variant GWAS and genomic prediction dissect the genetic architecture underlying tomato fruit weight
Source: Theor Appl Genet. 2026 Sep 5;139(9):259. doi: 10.1007/s00122-026-05326-2 (PMC13546169; doi:10.1007/s00122-026-05326-2)
Supplement: Supplementary file 1 [file 122_2026_5326_MOESM1_ESM.docx]

**Supplementary Fig. S1. Genome-wide Manhattan plots of SNP-based genome-wide association analyses for fw across four environments. Manhattan plots show the distribution of SNP associations for fw in a) Antalya, b) Valencia, c) Georgia, and d) Florida. The x-axis represents the physical positions of SNPs across the 12 tomato chromosomes, and the y-axis shows the association significance as −log₁₀(P). The solid green line indicates the Bonferroni significance threshold, whereas the dashed green line indicates the suggestive significance threshold. SNPs exceeding the Bonferroni threshold were considered significantly associated with fw.**

**Supplementary Fig. S2. Genome-wide Manhattan plots of INDEL-based genome-wide association analyses for fw across four environments. Manhattan plots show the distribution of INDEL associations for fw in a) Antalya, b) Valencia, c) Georgia, and d) Florida. The x-axis represents the physical positions of SNPs across the 12 tomato chromosomes, and the y-axis shows the association significance as −log₁₀(P). The solid green line indicates the Bonferroni significance threshold, whereas the dashed green line indicates the suggestive significance threshold. INDELs exceeding the Bonferroni threshold were considered significantly associated with fw.**

**Supplementary Fig S3. Genome-wide Manhattan plots of SV-based genome-wide association analyses for fw across four environments. Manhattan plots show the distribution of SV associations for fw in a) Antalya, b) Valencia, c) Georgia, and d) Florida. The x-axis represents the physical positions of SNPs across the 12 tomato chromosomes, and the y-axis shows the association significance as −log₁₀(P). The solid green line indicates the Bonferroni significance threshold, whereas the dashed green line indicates the suggestive significance threshold. SVs exceeding the Bonferroni threshold were considered significantly associated with fw.**

**Supplementary Fig. S4.** Quantile–quantile (Q–Q) plots of genome-wide association analyses for fruit weight across four environments using three variant types. Panels **a–d** show results based on SNP markers, **e–h** show results based on INDEL markers, and **i–l** show results based on structural variants (SVs). For each variant type, association analyses were conducted separately in Antalya (a, e, i), Florida (b, f, j), Georgia (c, g, k), and Valencia (d, h, l) using the BLINK model. The red diagonal line represents the expected distribution of test statistics under the null hypothesis, while blue points represent the observed −log10(P) values. Deviations from the expected distribution at the upper tail indicate significant marker–trait associations for fruit weight.

**Supplementary Fig. S5**. LD patterns surrounding significant SNP loci associated with fw in the tomato Varitome panel. Each panel shows an LD heatmap constructed using genome-wide HapMap SNP markers located within a 400-kb window centered on the significant SNP identified by GWAS. The red triangle indicates the focal SNP, and color intensity represents pairwise linkage disequilibrium (r²) between markers, ranging from 0 (dark blue) to 1 (yellow). Chromosome number, SNP position, and the genomic interval corresponding to the LD-defined region are indicated above each panel. LD intervals were determined by identifying the upstream and downstream genomic positions at which LD with the focal marker decayed below an r² threshold of 0.20. These heatmaps illustrate the extent of local LD surrounding significant fruit weight association signals across the tomato genome.

**Supplementary Fig S6. LD patterns surrounding significant INDEL loci associated with fw in the tomato Varitome panel.** Each panel shows an LD heatmap generated using polymorphic HapMap SNP markers surrounding a significant INDEL identified by GWAS. For most loci, LD was evaluated within a 400-kb genomic interval (±200 kb) centered on the focal INDEL. For the loci on chromosome 4 (13,734,554 bp) and chromosome 11 (31,865,292 bp), a 1-Mb genomic interval (±500 kb) was analyzed to better capture the local LD structure. The red triangle indicates the polymorphic HapMap marker nearest to the focal INDEL used for LD estimation, and color intensity represents pairwise LD (r^2^) between markers, ranging from 0 (dark blue) to 1 (yellow). Chromosome number, focal INDEL position, and the genomic coordinates of the corresponding LD interval are indicated above each panel. LD intervals were defined by the upstream and downstream genomic positions at which LD with the focal marker decayed below an r² threshold of 0.20. These heatmaps illustrate the extent of local LD surrounding significant fw association signals detected using INDEL markers. When LD did not decay below this threshold within the analyzed interval, the boundary was assigned to the outermost polymorphic marker in that direction.

**Supplementary Fig. S7. LD patterns surrounding significant SV loci associated with fw in the tomato Varitome panel.** Each panel shows an LD heatmap generated using HapMap SNP markers located within a 400-kb window centered on a significant SV identified by GWAS. The red triangle indicates the focal SV, and color intensity represents pairwise LD (r²) between markers, ranging from 0 (dark blue) to 1 (yellow). Chromosome number, focal SV position, and the genomic coordinates of the corresponding LD interval are indicated above each panel. LD intervals were defined by the upstream and downstream genomic positions at which LD with the focal marker decayed below an r² threshold of 0.20. These heatmaps illustrate the extent of local LD surrounding significant fw association signals detected using SV markers.

**Supplementary Fig. S8. Effect of linkage disequilibrium (LD) pruning on marker density and genomic prediction accuracy. a) Number of SNPs retained after LD pruning at pairwise r^2^ thresholds ranging from 0.1 to 0.9. b) Mean prediction accuracy of PLS regression using genomic relationship matrices constructed from the LD-pruned marker sets. Prediction accuracy was calculated as the Pearson correlation between observed and predicted fruit weight BLUPs. Values within each cell represent the mean correlation ± standard deviation across 100 random train–test partitions under four cross-validation scenarios. CV1 and CV2 represent prediction in tested environments for training and testing pedigrees, respectively, whereas CV0 and CV00 represent prediction in the omitted environment for training and testing pedigrees, respectively.**

**Supplementary Fig. S9. Root mean square error (RMSE), mean absolute error (MAE), and prediction bias of genomic prediction models under four cross-validation (CV) scenarios. Heatmaps show the mean ± SD across 100 random train–test partitions for four prediction algorithms (BGLR, PLS, RF, and DL) evaluated under four CV scenarios (CV1, CV2, CV0, and CV00) and five genomic prediction models: M1, SNPs; M2, INDELs; M3, SVs; M4, SNP + INDEL + SV; and M5,** M4 + G×E **kernels. Panels show a) root mean square error (RMSE), b) mean absolute error (MAE), and c) prediction bias. Lower RMSE and MAE values indicate better prediction performance, whereas prediction bias values closer to zero indicate lower systematic prediction error.**
